# Culture and amplification-free nanopore sequencing for rapid detection of pathogens and antimicrobial resistance genes from urine

**DOI:** 10.1007/s10096-024-04929-1

**Published:** 2024-09-16

**Authors:** Anurag Basavaraj Bellankimath, Crystal Chapagain, Sverre Branders, Jawad Ali, Robert C Wilson, Truls E. Bjerklund Johansen, Rafi Ahmad

**Affiliations:** 1 Department of Biotechnology, Inland Norway University of Applied Sciences, Holsetgata 22, Hamar, 2317 Norway; 2 Institute of Clinical Medicine, University of Oslo, Oslo, Norway; 3 Institute of Clinical Medicine, University of Aarhus, Aarhus, Denmark; 4 Institute of Clinical Medicine, Faculty of Health Sciences, UiT - The Arctic University of Norway, Hansine Hansens veg 18, Tromsø, 9019 Norway

**Keywords:** Metagenomic whole genome sequencing, Urinary tract infections, Culture free sequencing, Antimicrobial resistance, Urine

## Abstract

**Purpose:**

Urinary Tract Infections (UTIs) are among the most prevalent infections globally. Every year, approximately 150 million people are diagnosed with UTIs worldwide. The current state-of-the-art diagnostic methods are culture-based and have a turnaround time of 2–4 days for pathogen identification and susceptibility testing.

**Methods:**

This study first establishes an optical density culture-based method for spiking healthy urine samples with the six most prevalent uropathogens. Urine samples were spiked at clinically significant concentrations of 10^3^-10^5^ CFU/ml. Three DNA extraction kits (BioStic, PowerFood, and Blood and Tissue) were investigated based on the DNA yield, average processing time, elution volume, and the average cost incurred per extraction. After DNA extraction, the samples were sequenced using MinION and Flongle flow cells.

**Results:**

The Blood and Tissue kit outperformed the other kits based on the investigated parameters. Using nanopore sequencing, all the pathogens and corresponding genes were only identified at a spike concentration of 10^5^ CFU/ml, achieved after 10 min and 3 hours of sequencing, respectively. However, some pathogens and antibiotic-resistance genes (ARG) could be identified from spikes at 10^3^ colony formation units (CFU/mL). The overall turnaround time was five hours, from sample preparation to sequencing-based identification of pathogen ID and antimicrobial resistance genes.

**Conclusion:**

This study demonstrates excellent promise in reducing the time required for informed antibiotic administration from 48 to 72 h to five hours, thereby reducing the number of empirical doses and increasing the chance of saving lives.

**Supplementary Information:**

The online version contains supplementary material available at 10.1007/s10096-024-04929-1.

## Introduction

Urinary Tract Infections (UTIs) are the second most prevalent infections worldwide, accounting for 1–3% of primary healthcare visits and contributing to around 13.7% of community-based antibiotic prescriptions [1, 2]. In 2019, there were 405 million cases of UTI globally, with 236,790 associated deaths, which is a 60% rise in cases and a 140% rise in deaths since 1990 [3, 4]. Untreated UTIs can progress to pyelonephritis, a more severe kidney infection, with pregnant women facing heightened risks of preterm delivery and low birth weight babies [5]. Pyelonephritis and uroepithelial invasion of pathogens can progress to urosepsis, which accounts for a quarter of all sepsis cases [6]. If not promptly treated, around 40% of hospital-acquired UTIs might cause severe conditions such as pyelonephritis and urosepsis [7], posing a significant risk to immunocompromised, catheterized, and elderly patients. In cases of severe UTIs, administering effective antibiotics as soon as possible is paramount. Due to a recent global increase in antimicrobial resistance (AMR) among urinary tract pathogens, fewer antibiotics are available as reliable clinical treatments.

UTIs are mainly caused by gastrointestinal bacteria, while fungal infections are rare. The most common pathogens include *E. coli*, *Klebsiella pneumoniae*, *Proteus mirabilis*, *Pseudomonas aeruginosa*, *Enterococcus* spp., *Staphylococcus* spp., and *Candida* spp [8, 9]. In the clinical routine, a bacterial concentration of ≥ 10^5^ CFU/mL in urine is the accepted criterion for symptomatic UTI [8]. However, depending on the clinical type and complexity, such as in pregnant women and complicated UTIs, the clinically significant bacteriuria concentrations can be as low as 10^2^ CFU/mL [10, 11]. The current diagnostic methods usually need 24 h to identify the pathogen, with an additional 24–48 h to determine the antibiotic susceptibility [12]. Currently, techniques such as mass spectroscopy-based matrix-assisted laser desorption ionization-time of flight (MALDI-TOF) are routinely used for pathogen identification, along with nucleic acid-based amplification tests that can perform both identification and genotypic resistance detection [13, 14]. While MALDI-TOF can offer rapid microbial identification, it requires the pre-cultivation of the pathogens. It is also difficult to differentiate closely related bacterial species, thereby reducing identification specificity [15]. Meanwhile, conventional nucleic acid-based tests such as PCR are restricted to specific pathogens and lack unbiased comprehensive identification capabilities [14].

Culture-free metagenomic next-generation sequencing (mNGS) has the potential for rapid unbiased identification of pathogens (both mono and polymicrobial UTIs) at the strain level and their corresponding AMR genes, overcoming the limitations of the current diagnostics methods [16]. mNGS is also superior in identifying slow-growing pathogens and directly detecting AMR genes without a targeted assay [16]. A study in which the mNGS using the Ion Torrent sequencing method was applied directly to clinical urine showed concordance with the conventional screening methods [17]. Schmidt et al. identified pathogens and antibiotic-resistance genes in clinical and spiked urine samples with a rapid turnaround time of 4–6 h [18]. Similarly, Zhang et al. optimized a metagenomic nanopore sequencing-based method and tested it on 76 patient samples, showing a detection sensitivity of 86.7% (95% CI) and a specificity of 96.8% (95% CI) compared to conventional detection methods [19]. Moreover, the introduction of Nanopore sequencing (Oxford Nanopore Technologies (ONT), UK) has dramatically reduced the pathogen detection time and sequencing costs, thereby exhibiting an excellent potential for rapid point-of-care testing [20]. The MinION is a pocket-sized USB-connectable, portable device capable of real-time long-read sequencing and data analysis [21, 22]. DNA extraction is a critical step for metagenomics-based diagnostic methods. Studies have been conducted to determine the efficiencies of different extraction kits on samples such as stool and blood [23–25]. However, efficient methods of bacterial DNA extraction from urine are seldomly explored due to challenges associated with urine, such as low microbial biomass, the presence of extraction interfering phosphate or urate salts, and the presence of different assay inhibitors such as beta-human chorionic gonadotropin and nitrites [26, 27].

The current work is a proof-of-concept study aimed at investigating the limit of detection (LOD) of bacterial pathogens in urine by establishing a culture and amplification-independent methodology of extracting DNA from spiked urine samples followed by nanopore sequencing. To achieve this, we first established an absorbance (A600) based culture method for preparing spiked urine samples at clinically relevant concentrations of 10^2^ to 10^5^ CFU/mL using the most prevalent uropathogens. It was followed by testing three commercial DNA extraction kits: QIAamp BiOstic Bacteremia DNA Kit, DNeasy Blood and Tissue Kit, and PowerFood Microbial Kit based on the DNA yield, average processing time, elution volume, and the average cost incurred per extraction. The extracted DNA was then sequenced, followed by real-time data analyses to identify and detect pathogens and reference ARGs. The results show that PCR could detect the presence of bacterial DNA in all samples with spike concentrations of ≥ 10^2^ CFU/mL, except for *S. aureus* samples. All the pathogens and their corresponding ARGs were identified at 10^5^ CFU/mL within 10 to 200 min of MinION sequencing. The overall Turn-Around-Time (TAT), including DNA extraction, library preparation, nanopore sequencing, and bioinformatic analysis, was 5 hours. Moreover, *E. coli* NCTC13441, *K. pneumoniae* CCUG225T, and *P. mirabilis* CCUG2676T were identified at 10^3^ CFU/mL, highlighting the method´s potential to detect pathogens at lower LOD. Such a culture- and amplification-independent workflow that combines real-time sequencing and data analysis could be transformative for clinical management of severe UTIs.

## Methods

### Experimental design

**Fig. 1:**
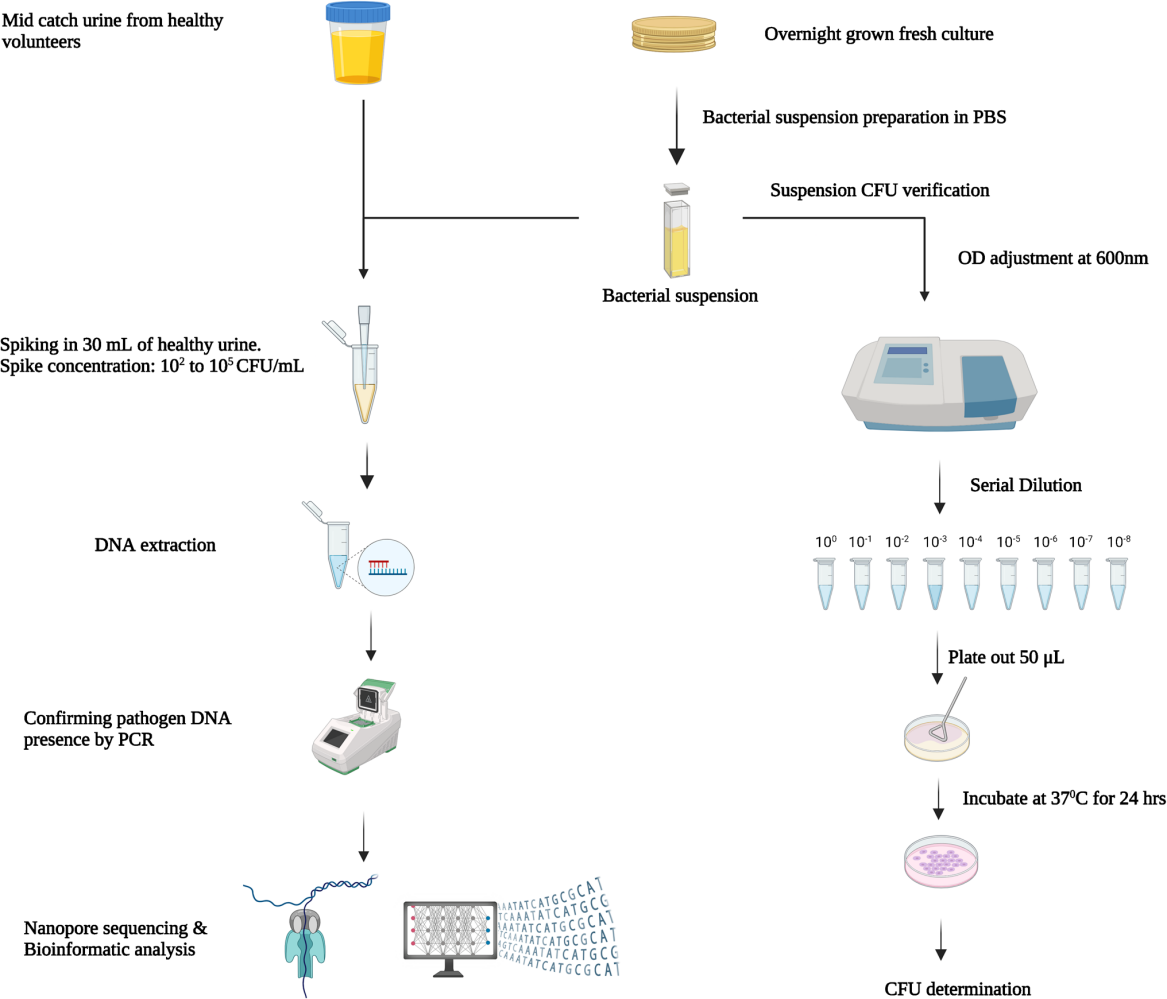
A graphical overview of the inoculum preparation, spiking, DNA extraction and sequencing steps involved in the study

Healthy urine samples were spiked with uropathogens at clinically relevant concentrations (10^2^ − 10^5^ CFU/mL) to imitate clinically relevant UTIs. DNA was extracted using the best-performing kit combined with an additional pre-processing step. PCR was used to verify the presence of the spiked species before sequencing using MinION or Flongle flow cells (Fig. 1). In addition, PCR confirmation was performed on extracts from a range of bacterial concentrations (10^2^ − 10^5^ CFU/mL) to check the efficiency of the DNA extraction methods.

#### Inoculum optimization of the bacterial strains relevant to UTI

The current study used nine strains representing six clinically relevant bacterial species commonly found in UTIs, namely *E. coli*,* K. pneumoniae*,* P. mirabilis*,* Enterococcus faecalis*,* P. aeruginosa*,* and S. aureus* (Supplementary Table 1). Four strains of *E. coli* were included in the study, as most UTI cases are associated with *E. coli*. Two *E. coli* isolates (*E. coli* NCTC 13441 *and E. coli* INN 2) and the *K. pneumoniae* isolates carried the extended-spectrum β-lactamases (ESβL) genes. The strains *K. pneumoniae* CCUG 225T, *E. faecalis* CCUG 9997, and *P. aeruginosa* CCUG 17619 were retrieved from the Culture Collection at the University of Gothenburg (CCUG, Sweden). *E. coli* NCTC 13441 and methicillin-sensitive strain *S. aureus* NCTC8325 were retrieved from the National Collection of Type Cultures (NCTC, UK Health Security Agency, UKHSA, UK). Three clinical strains (*E. coli* INN 1, *E. coli* INN 2, and *E. coli* INN 3) were used from our in-house collection at INN University, Hamar.

The bacterial strains were revived by streaking a loopful of frozen glycerol stock on Brain Heart Infusion (BHI) agar plates (15 g/L Agar, 37 g/L BHI Broth, VWR Life Science, USA) followed by incubation at 37 ± 2 °C overnight. Individual colonies from the overnight culture were resuspended in 1X phosphate buffered saline (PBS) (E504-100ML, VWR Life Science, USA), and the absorbance of bacterial suspensions at an optical density of 600 nm (A600) was measured using a UV 3100PC Spectrophotometer (VWR Life Science, USA). The A600 of the prepared suspensions were then adjusted to 10^9^ or 10^8^ CFU/mL depending on the target bacterial species (Supplementary Table 2). To verify the CFU, the prepared suspension of known A600 was serially diluted up to 10^−6^ dilutions, and 50 µL was spread on BHI agar plates and incubated at 37 ± 2 °C for 24 h [24]. Further experiments were only carried out when the spiked inoculum concentration was in the range of target CFU/mL (10^9^ or 10^8^). This suspension was then used to spike healthy urine samples at final concentrations of 10^2^ to 10^5^ CFU/mL. Four to seven suspension replicates were prepared for all the bacterial strains used in the study to determine the correlation between the A600 and CFU/mL (Supplementary Table 2). The A600 was used as a starting reference point, and precise viable bacteria numbers were determined using the plate count method (Supplementary Fig. 1).

#### Evaluation of DNA extraction kits

Three commercial DNA extraction kits from Qiagen Germany, including the QIAamp BiOstic Bacteremia DNA Kit, DNeasy Blood and Tissue Kit, and PowerFood Microbial Kit, were used and compared based on DNA yield, average processing time, and cost per sample. The kits were tested on two gram-negative bacteria (*E. coli* and *K. pneumoniae*) and one gram-positive bacterium (*S. aureus*) at 10^2^ to 10^5^ CFU/mL. In addition, for the Blood and Tissue kit, the gram-positive (enzymatic lysis buffer containing 20 mg lysozyme, 20 mM Tris. Cl, 2mM Sodium EDTA & 1.2% Trition-X-100) and gram-negative bacteria pretreated as per the manufacturer’s instructions. Finally, the best-performing kit was selected for the direct DNA extraction steps.

### PCR-based identification of spiked pathogenic DNA

The presence of bacterial DNA in the extracted DNA was verified using species-specific PCR primers (Supplementary Table 4). PCR amplification was performed using 20 µl reaction mixtures containing 4 µl of 5 X HOT FIREPol^®^ MultiPlex Mix Ready to Load with 10 mM MgCl_2_ (Solis BioDyne, Estonia), 0.5 µL of 10 µM forward and reverse primers and 1–8 ng template DNA (nucleic acid- and nuclease-free water for negative controls). PCR was performed using a Verity 96-Well Thermal Cycler (Applied Biosystems, USA), with the following thermal profile: initial denaturation for 12 min at 95 °C followed by 30 cycles of 25 s at 95 °C, 45 s at 60 °C and 45 s at 72 °C, with final extension at 72 °C for 7 min. Results were visualized using 1% agarose gel electrophoresis using G: box (SYNGENE, USA).

### Direct DNA extraction from spiked urine samples and sequencing

For preparation of spiked urine samples, a 30 ml clean catch midstream urine sample from a healthy donor was collected in a sterile bottle as described by Cheesbrough et al. [28]. The prepared bacterial suspension with 10^9^/10^8^ CFU/mL was used to spike 30 mL of collected urine sample to the final desired concentrations of 10^2^ to 10^5^ CFU/mL. Moreover, a control experiment was conducted, where non-spiked (healthy urine) and spiked urine samples were cultured on MacConkey agar to determine the presence of commensal microflora and contaminants. The spiked samples were then pre-processed by the addition of 10% (v/v) of 1 M Tris-EDTA (10 mM Tris, 1 mM EDTA, pH 8.0) and centrifuged at 3260*g* for 10 min at 4 °C as described by Munch M M et al., [29]. The resulting pellet was used for further DNA extraction by the best-performing extraction kit (Sect. 2.1.2). All the direct extractions were performed in duplicate. After extraction, the concentrations were determined using the Qubit dsDNA HS Assay kit (ThermoFisher Scientific), after which PCR was used to verify the presence of bacterial DNA, as described in Sect. 2.2. The samples with concentrations of 10^3^ and 10^5^ CFU/mL were sequenced on MinION flow cells. The Agencourt AMPure XP system (Beckman Coulter, USA) was used for DNA purification, and library preparation for MinION sequencing was performed using the Rapid Barcoding Sequencing kit SQK-RBK004 (Oxford Nanopore, UK) according to the respective manufacturer’s instructions. The library was sequenced using MinION flow cells (R9.4.1 FLO-MIN106D, Oxford Nanopore) for 18 h, after which the flow cell was washed and reused for the rest of the samples.

In addition, three pathogens (*E. coli* INN 1, *E. faecalis* CCUG 9997, and *P. aeruginosa* CCUG 17619) were sequenced on Flongle flow cells. Five ml spiked urine samples were prepared for each pathogen at 10^3^, 10^4,^ and 10^5^ CFU/mL spike concentrations. DNA extraction, purification, and concentration were performed as mentioned above. The library was made using the Ligation Sequencing Kit (SQK-LSK109) and Native Barcoding Expansion Kit (EXP-NBD104) according to the manufacturer’s instructions and sequenced using Flongle flow cells (R9.4.1 FLO-FLG001, Oxford Nanopore).

### Bioinformatic analysis of the sequencing data

Sequencing was performed with default parameters. The quality score (q-score) was set to ≥ 8. The data was basecalled in real-time using the MinKNOW software (version 5.3.1) having Guppy basecaller (version 6.3.9). Pathogen identification, ARG detection, and Genome Coverage analysis were performed using in-house developed pipelines; identification of pathogens was performed using BLASTn [30] (version 2.14.0) against the NCBI prokaryotic reference (RefProk) collection [16]. ARGs were detected using the ABRicate tool (version 1.0.1) (Seemann T, Abricate, Github https://github.com/tseemann/abricate) with the CARD [31], ResFinder [32], MEGARES [33] and NCBI AMRFinderPlus [34] databases. Genome coverage was obtained by BLASTn, equivalent to Taxt et al., 2020 [25].

Unless stated, all the graphs and figures used in this study were created using BioRender, GraphPad Prism (10.0.2), and RStudio (1.4.1106) software.

## Results

### Establishment of the optimal experimental conditions for mimicking clinically relevant urine samples

A600 provided a preliminary estimate of the bacterial concentration for spiking the urine samples. The protocol optimization experiments were initially conducted using only the wild-type *E. coli* CCUG 17620 strain. The results showed that A600 data for suspensions between OD 1.2–1.8 corresponded to cell concentrations ranging from 2.1*10^8^ − 2.1*10^9^ CFU/mL (Supplementary Table 2). Although OD provides a preliminary assessment of cell concentration, it does not necessarily correlate precisely to viable bacteria. Hence, the prepared bacterial suspensions were always plated out to determine the exact CFU/mL.

To investigate whether DNA extraction directly after spiking is comparable to extraction from clinical urine infection samples, the CFU was calculated directly after spiking and after one hour of acclimatization to the urine environment for each species. The results indicate no difference in CFU before and after one hour of spiking, indicating that the targeted bacterial load is reached directly after spiking (Supplementary Table 3). Additionally, culturing non-spiked healthy urine samples on MacConkey agar didn’t show any bacterial growth, indicating the absence of any significant background microflora (Supplementary Fig. 2).

### Blood and Tissue kit yields a higher concentration of DNA than the other kits

**Fig. 2:**
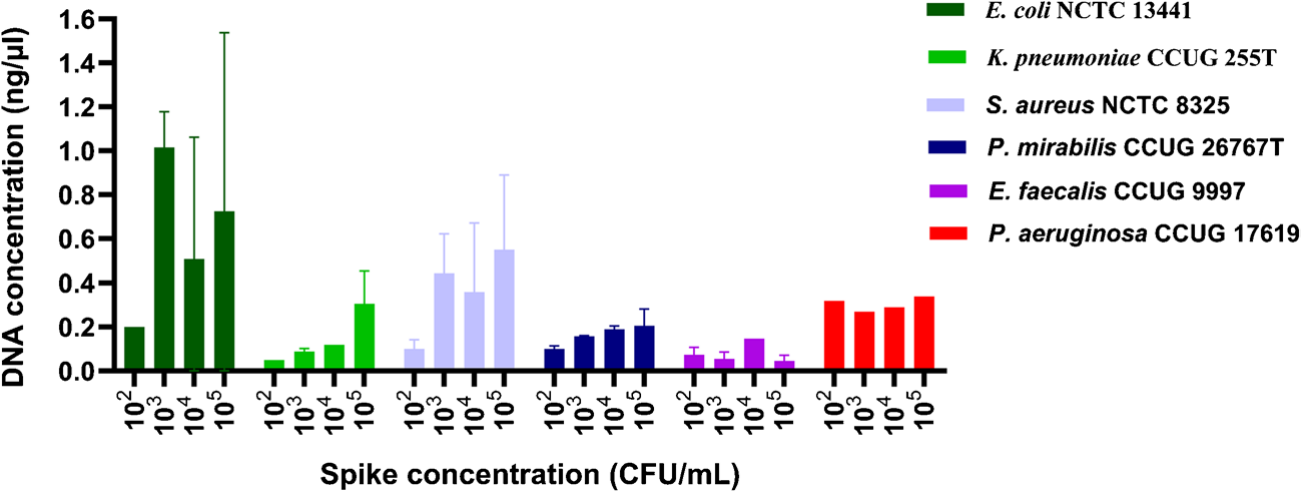
Comparison of DNA concentrations in extracts from urine samples spiked with different species. The DNA was extracted from 30 ml urine samples spiked with 10^2^ to 10^5^ CFU/mL of the target bacteria using a Blood and Tissue kit. The DNA concentration was measured using Qubit. All the extractions were performed in duplicates except for *P. aeruginosa* CCUG 17619, where a single extraction was performed. The DNA concentration for the three in-house strains*, E. coli* INN 2*, E. coli* INN 1*, and E. coli* INN 3*,* is presented in Supplementary Fig. 4

The performance of three commercial kits, BiOstic, PowerFood, and Blood and Tissue, was tested on two gram-negative (*E. coli* & *K. pneumoniae*) and one gram-positive (*S. aureus*) species. The comparison was based on DNA yield, average processing time, and cost per sample to select the best kit. The obtained concentrations are summarised in Table 1. On average, all the extracted DNA concentrations from all three kits were > 0.9 ng/µL. However, the Blood and Tissue kit outperformed the other kits in all three parameters, having higher DNA yields, lower average processing time (35 min), and cost per extraction (3.94 €). The Blood and Tissue kit was used for downstream direct DNA extraction steps, the results of which are summarised in Fig. 2. Overall, higher yields were obtained from the samples spiked with *E. coli* NCTC 13441, while *E. faecalis* CCUG 9997 had the lowest yield. Moreover, pathogen DNA was detected by PCR among samples spiked with ≥ 10^2^ CFU/mL, except for *S. aureus* samples, where the detection was possible only at 10^4^ and 10^5^ CFU/mL (Supplementary Fig. 3).

**Table 1:** Comparison of DNA concentration isolated using three different kits. The DNA yield of BiOstic, PowerFood, and DNeasy Blood and Tissue kits was compared using two gram-negative and one gram-positive bacteria. In the case of *S. aureus*, the DNA was isolated using two different strains (*S. aureus CCUG 17621* for BiOstic and *S. aureus NCTC 8325* for PowerFood and Blood and Tissue). NA indicates no DNA was isolated

	BiOstic (ng/μl)				Powerfood (ng/μl)				DNeasy Blood and Tissue (ng/μl)			
	10^2^ CFU/mL	10^3^ CFU/mL	10^4^ CFU/mL	10^5^ CFU/mL	10^2^ CFU/mL	10^3^ CFU/mL	10^4^ CFU/mL	10^5^ CFU/mL	10^2^ CFU/mL	10^3^ CFU/mL	10^4^ CFU/mL	10^5^ CFU/mL
*E. coli*	0.14	< 0.05	0.08	0.48	0.54	< 0.05	< 0.05	0.38	0.2	0.9	0.9	1.3
*K. pneumoniae*	0.2	< 0.05	0.06	0.67	0.29	< 0.05	0.09	0.47	< 0.05	0.10	0.12	0.41
*S. aureus*	0.4	0.75	0.64	NA	0.334	0.31	0.37	0.33	0.072	0.57	0.58	0.79
Elution Volume (μl)	50				100				200			
Average processing Time (min)	60				45				35			
Average cost per sample (€)	4.64				4.29				3.94

### Direct nanopore sequencing using MinION flow cells

**Fig. 3:**
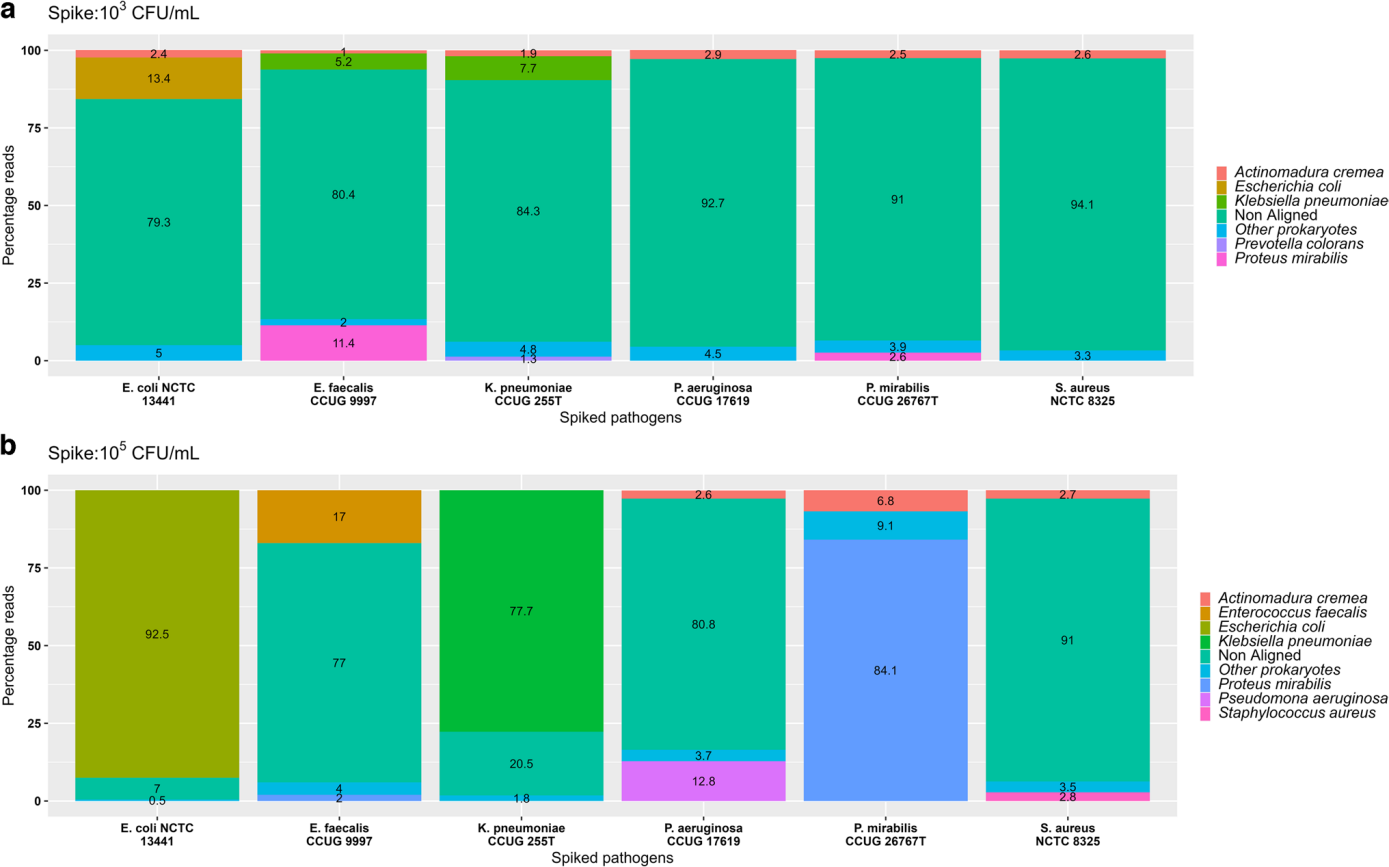
Relative distribution of the reads generated by MinION sequencing of spiked urine samples. The tabulated results are from all the reads generated by the MinION platform from the sequencing start. Subfigures (**a**) and (**b**) represent the samples spiked at 10^3^ and 10^5^ CFU/mL, respectively. The denoted percentage reads are based on the BLAST search against the RefProk database (prokaryotic sequence data only). The data for the rest of the three in-house strains, *E. coli* INN 2, *E. coli* INN 1, and *E. coli* INN 3, is presented in Supplementary Fig. 5

The sequencing run generated 7.06 M reads with 11.24 gigabases (Gb), including 89% of passed reads with an estimated N50 of 3.6 kb. The q-score of the sequencing run ranged from 9 to 13, with an average value of 11.5. All the sequence reads generated from the MinION flow cells from each sample were searched against the NCBI RefProk database using BLASTn. The results for *E. coli* NCTC 13441, *K. pneumoniae* CCUG 225T, *P. aeruginosa* CCUG 17619, *S. aureus* NCTC 8325, *E. faecalis* CCUG 9997, and *P. mirabilis* CCUG 2676T are summarised in Table 2; Fig. 3. All the bacteria were successfully identified at 10^5^ CFU/mL. Interestingly, pathogen identification was also possible at 10^3^ CFU/mL for samples spiked with *E. coli* NCTC 13441, *K. pneumoniae* CCUG 225T, and *P. mirabilis* CCUG 2676T.

**Table 2:** Summary of the pathogen identification and antimicrobial resistance gene detection by MinION nanopore sequencing from two different concentrations of spiked urine samples. The order of the organisms is based on their prevalence in complicated UTIs. Green ticks: positive identification; red cross are unidentified for pathogen identity and antibiotic resistant genes

Strains	CFU/mL	DNA (ng/μl)	Pathogen Id	ARG
*E. coli* NCTC13441	10^3^	0.90	**✔**	**✔**
	10^5^	1.30	**✔**	**✔**
*E. coli* INN 1	10^3^	1.13	**✘**	**✘**
	10^5^	1.15	**✔**	**✘**
*E. coli* INN 2	10^3^	0.88	**✘**	**✘**
	10^5^	0.97	**✔**	**✔**
*E. coli* INN 3	10^3^	0.05	**✘**	**✘**
	10^5^	0.20	**✔**	**✔**
*E. faecalis* CCUG9997	10^3^	0.08	**✘**	**✘**
	10^5^	0.24	**✔**	**✘**
*K. pneumoniae* CCUG225T	10^3^	0.10	**✔**	**✘**
	10^5^	0.41	**✔**	**✔**
*P. mirabilis* CCUG2676T	10^3^	0.15	**✔**	**✘**
	10^5^	0.26	**✔**	**✔**
*P. aeruginosa* CCUG17619	10^3^	0.27	**✘**	**✘**
	10^5^	0.34	**✔**	**✔**
*S. aureus* NCTC 8325	10^3^	0.57	**✘**	**✘**
	10^5^	0.79	**✔**	**✔**

The initial BLAST search with the RefProk database misclassified 1–3% of reads as *Actinomadura cremea* in samples with *P. mirabilis*, *P. aeruginosa*, and *S. aureus*. However, a BLAST search against the human genome revealed that these reads were derived from human mitochondrial DNA, which shows errors in current public database annotations. The remaining identified bacterial species with less than 1% assigned reads were collectively presented as other prokaryotes. In the 10^5^ CFU/mL *E. coli* NCTC 13441 sample, 92.5% of the reads were assigned to *E. coli*, 7% were non-aligned (human reads), and 0.5% were other prokaryotes. In comparison, in the sample with 10^3^ CFU/mL of the same strain, the percentage of non-aligned reads increased from 7 to 80%, and the percentage of bacterial reads decreased from 92.5 to 13%. Similar results were also observed in samples spiked at 10^3^ and 10^5^ CFU/mL concentrations *K. pneumoniae* CCUG 225T (77% & 7.69%) and *P. mirabilis* CCUG 2676T (27% & 2.6%). In these three samples, the highest number of prokaryotic reads were assigned to the reference pathogen spike, confirming the identification at both concentrations.

For samples spiked with *P. aeruginosa* CCUG 17619 and *S. aureus* NCTC 8325, the percentage of non-aligned reads was 92.7% and 94.1% at 10^3^ CFU/mL and decreased at 10^5^ CFU/mL concentration to 80.8% and 91%, respectively. Also, 12.8% and 2.8% of the respective sequencing reads at 10^5^ CFU/mL were assigned to *P. aeruginosa* and *S. aureus*. However, no bacterial reads from these two species were detected at 10^3^ CFU/mL. The sample spiked with 10^5^ CFU/mL *E. faecalis* CCUG 9997 contained 77% non-aligned reads, 16% *E. faecalis*, 3% *P. mirabilis*, 1% *Actinomadura cremea*, and 3% other prokaryotes. However, at 10^3^ CFU/mL, the obtained reads comprised 79% non-aligned, 12% *P. mirabilis*, 6% *K. pneumoniae*, and 3% other prokaryotes. Thus, the target pathogen, *E. faecalis*, was only identified in the sample spiked with 10^5^ CFU/mL.

#### Detection of antibiotic resistance genes

**Fig. 4:**
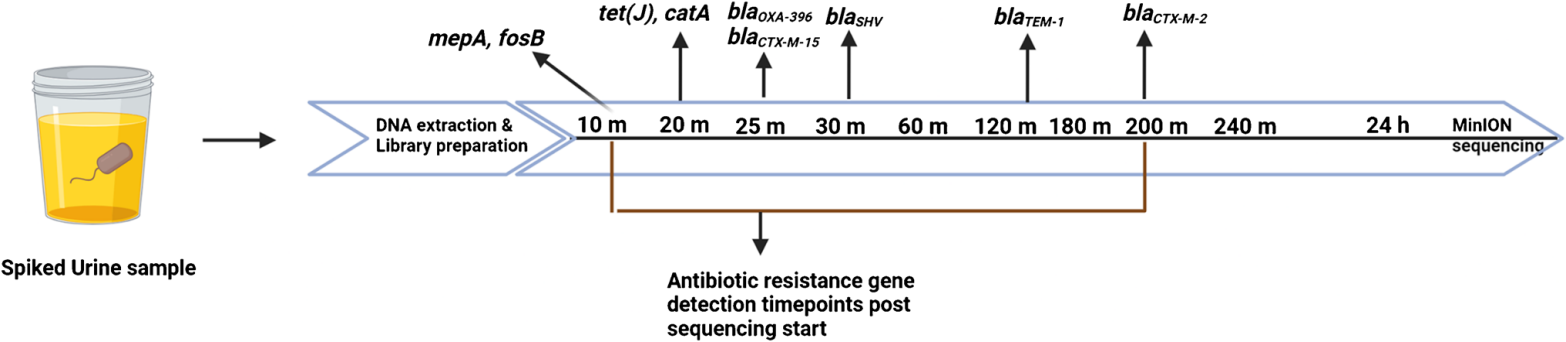
Detection of reference AMR genes over time for samples spiked with 10^5^ CFU/mL. Most of the genes were detected within 30 min of sequencing except bla_*TEM-1*_ and bla_*CTX-M-2*_, which took 140 and 200 min, respectively. Bla_*CTX-M-15*_ was detected after 13 h of sequencing from the sample spiked with 10^3^ CFU/mL

Of the nine samples spiked with 10^5^ CFU/mL, reference AMR genes were detected in all except *E. faecalis* CCUG9997 and *E. coli* INN 1. In 10^3^ CFU/mL samples, the detection was possible only in the *E. coli* NCTC 13441 sample (Table 2). The reference AMR gene *bla*_*CTX−M−15*_ present in *E. coli* NCTC 13441 was detected in urine samples at 10^3^ and 10^5^ CFU/mL concentrations. In the 10^5^ CFU/mL sample, this gene was detected after 25 min, but it took as long as 13 h to detect it in the 10^3^ CFU/mL sample. For *E. coli* INN 2, the target AMR gene, *bla*_*CTX−M−2*_, was detected within 200 min. For *E. coli* INN 3, the target AMR gene *bla*_*TEM−1*_ was detected in 137 min. For *K. pneumoniae*, seven variants of the target gene *bla*_*SHV*_ were detected, but the specific variant of interest, *bla*_*SHV−11*_, was not identified. In the case of *P. mirabilis* CCUG 2676T, both the reference genes, *catA* and *tet(J)*, were detected within 20 min of sequencing, while for *P. aeruginosa* CCUG 17619, the target gene *bla*_*oxa−356*_ was detected within 25 min. For *S. aureus* NCTC 8325 samples, *fosB* and *mepA* genes were detected within 10 min, along with *mepB* and *mepR*, which form the *mepRAB* gene cluster. Finally, no target ARGs were identified for *E. coli INN 1* and *E. faecalis* CCUG 9997 samples at either tested concentration. The reference AMR genes were detected between 10 – and 200 min in all the samples. A summarised detection timeline is presented in Fig. 4.

#### Genome coverage analysis at different concentrations of spiking

**Fig. 5:**
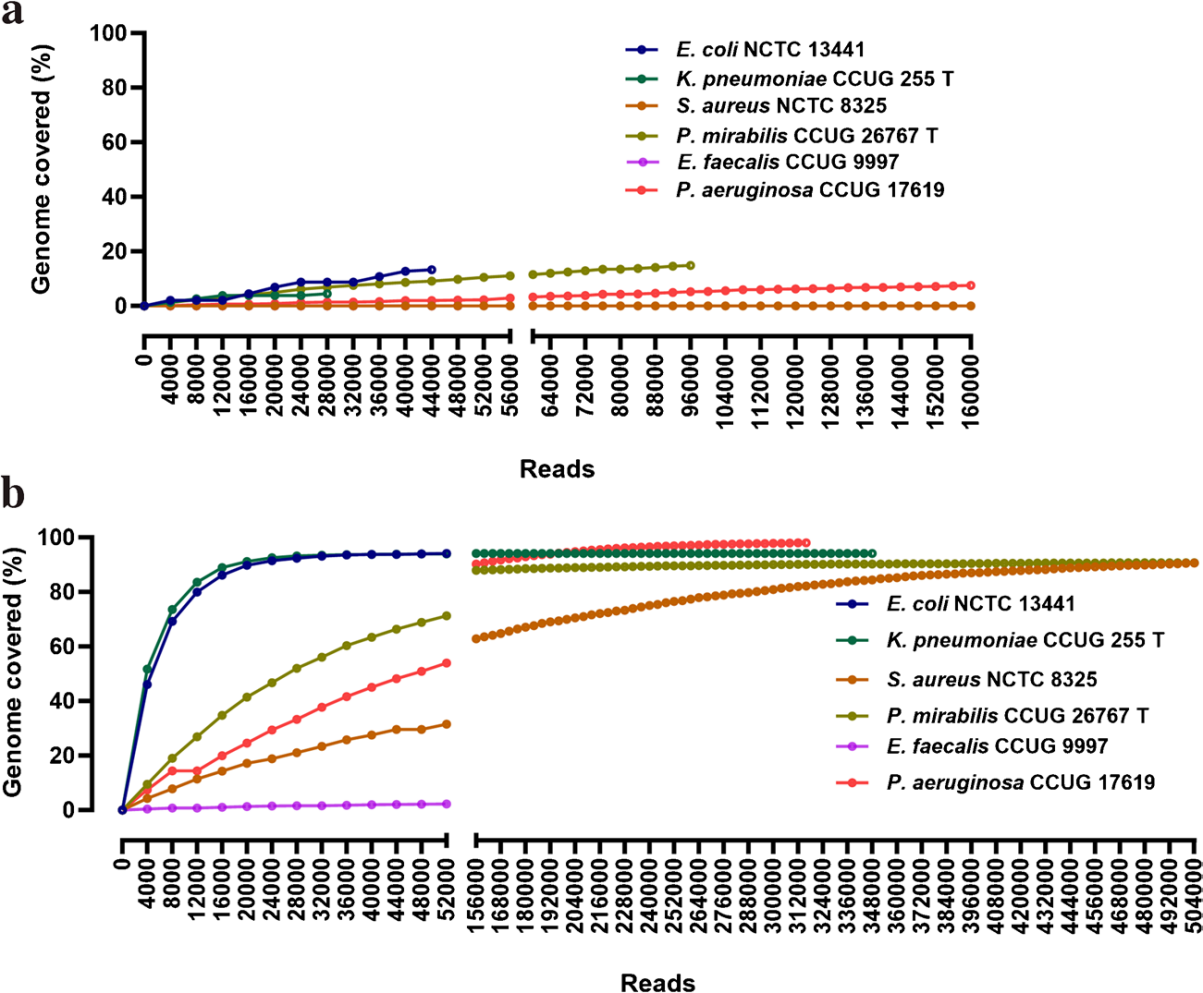
Genome coverage of the target species based on the sequencing reads generated during the sequencing run. The subfigure (**a**) represents the pathogen coverage obtained for the samples spiked with 10^3^ CFU/mL concentration, while the subfigure (**b**) is the pathogen coverage from the samples spiked with a pathogen concentration of 10^5^ CFU/mL

The raw sequencing reads obtained per bin were aligned to the reference genome of the respective species to get information regarding genome coverage over time (Fig. 5). Among the species spiked with 10^5^ CFU/mL, the genome coverage was > 90% for five samples, including *E. coli* NCTC 13441, *K. pneumoniae* CCUG 255T, *P. mirabilis* CCUG 26767T, *P. aeruginosa* CCUG 17619, and *S. aureus* NCTC 8325 (Fig. 5b). For 10^3^ CFU/mL samples, the genome coverage for gram-negative species, *E. coli* NCTC 13441, *K. pneumoniae*, *P. mirabilis*, and *P. aeruginosa*, was around 10%. However, the coverage of gram-positive *E. faecalis* and *S. aureus* was < 1%. The genome coverage of three in-house *E. coli* strains at 10^5^ CFU/mL was 25.7% for INN 1, 37.6% for INN 2, and 7% for INN 3. The genome coverage at 10^3^ CFU/mL was below 5% for these in-house *E. coli* strains (Supplementary Fig. 6).

### Direct nanopore sequencing using flongle flow cells

**Fig. 6:**
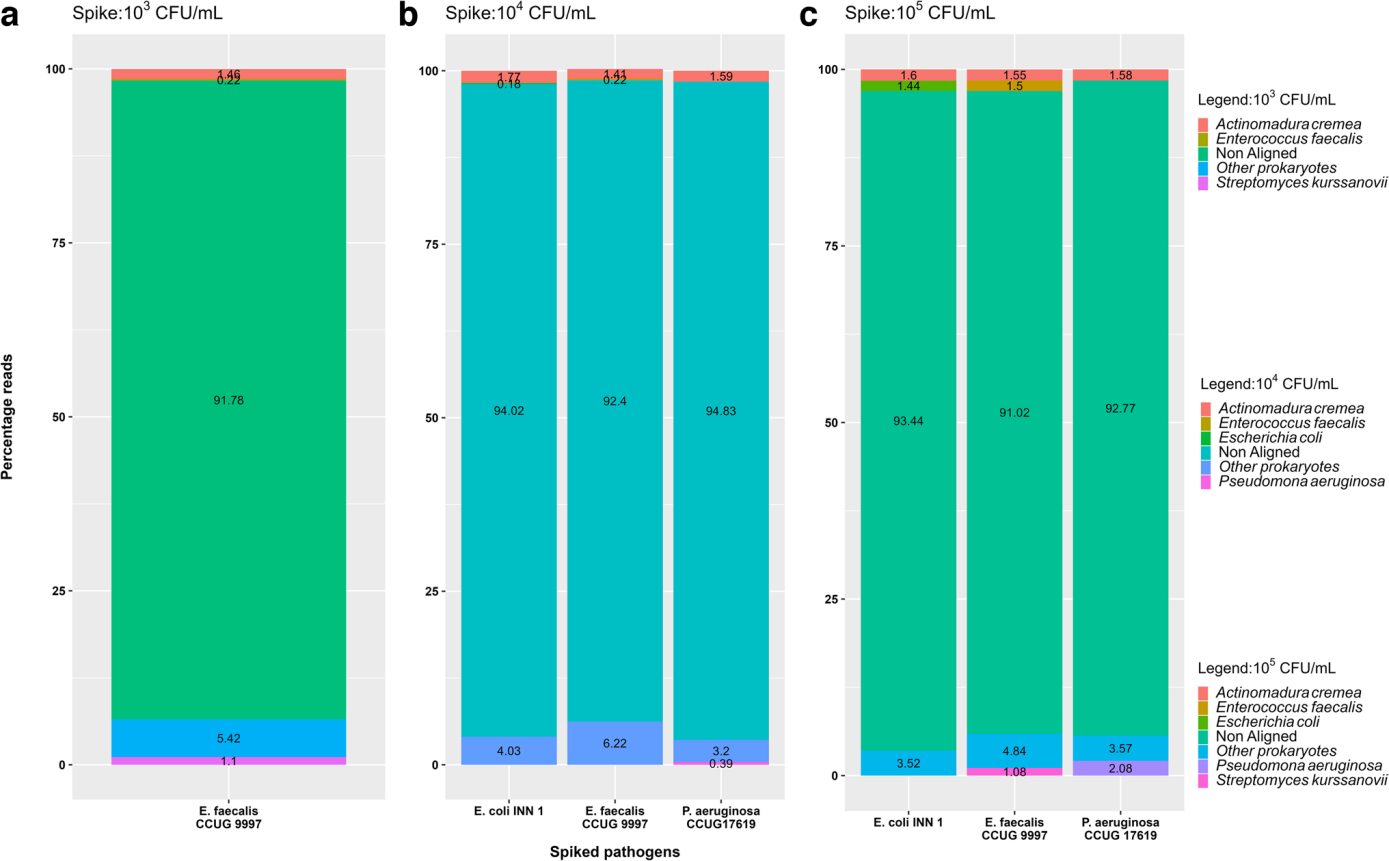
Relative distribution of reads generated by nanopore sequencing of DNA purified from spiked urine samples on Flongle flow cells. Subfigures (**a**), (**b**), and (**c**) represent the samples spiked at 10^3^, 10^4^, and 10^5^ CFU/mL, respectively. The represented percentage reads are based on a BLAST search against the RefProk database (prokaryotic sequence data only). The bacterial species (except the spiked pathogen) that were identified and had less than 1% of the total assigned reads were grouped and represented as "other prokaryotes."

For Flongle sequencing data, all the reads were considered to identify the pathogen and ARGs. At 10^5^ CFU/mL, 1.4% of *E. coli*, 1.5% of *E. faecalis*, and 2.1% of *P. aeruginosa* reads were identified. In all these three samples, the highest number of prokaryotic reads were found to be from the reference pathogen spike. Of reads from spiked samples at 10^4^ CFU/mL, only 0.2% with *E. coli*, 0.2% with *E. faecalis*, and 0.4% with *P. aeruginosa* mapped to the reference bacteria. The samples spiked with 10^3^ CFU/mL of *P. aeruginosa* CCUG 17619, and *E. coli* INN 1 didn’t yield detectable DNA after direct extraction using the Blood and Tissue kit. Also, no reads were assigned to *E. faecalis* at 10^3^ CFU/mL. Similar to the MinION result, 1–3% of reads in all the samples were misclassified as *Actinomadura cremea*, highlighting potential errors in current public database annotations. All three sequenced pathogens (*E. coli*, *P. aeruginosa*, and *E. faecalis*) were identified only at 10^5^ CFU/mL (Table 3; Fig. 6). Moreover, none of the target ARGs were identified at any of the three concentrations (10^3^, 10^4^, and 10^5^ CFU/ml) using the Flongle flow cell.

**Table 3:** Summary of the Pathogen Identification and AMR gene detection by Flongle sequencing from three different concentrations of 5 mL spiked urine samples. The legend for the figure is as follows: green tick: positive identification, red cross: unidentified, and NA: no quantifiable DNA was isolated, and samples were excluded from the sequencing

Spiked pathogens	CFU/mL	DNA (ng/μl)	Pathogen Id	ARG
*E. faecalis* CCUG9997	10^3^	0.100	**✘**	**✘**
	10^4^	0.120	**✘**	**✘**
	10^5^	0.160	**✔**	**✘**
*P. aeruginosa* CCUG17619	10^3^	NA	**-**	**-**
	10^4^	0.300	**✘**	**✘**
	10^5^	0.304	**✔**	**✗**
*E. coli* INN 1	10^3^	NA	**-**	**-**
	10^4^	0.270	**✘**	**✘**
	10^5^	0.310	**✔**	**✘**

## Discussion

Rapid and accurate pathogen and antibiotic resistance detection is critical for effective clinical management of UTI and corresponding antibiotic therapy. This proof-of-concept study aimed to establish a fast, culture-and-amplification-free method of detecting pathogens and their corresponding ARGs through nanopore sequencing from spiked urine samples. The current study established an A600 and CFU/mL-based method for spiking healthy urine samples to mimic clinical samples and establishing a pipeline for direct DNA extraction from these spiked samples. Three commercial DNA isolation kits were analyzed, and the Blood and Tissue kit outperformed the other kits based on the DNA yield, length of extraction, and cost per sample. At 10^5^ CFU/mL, all the pathogens were identified by nanopore sequencing within 1.5 h following sample collection. The first 4000 reads generated within 10 min of sequencing start were enough for pathogen identification, while three hours of sequencing was required for ARG detection. Therefore, the TAT from sample collection to identifying pathogens and ARGs, including sample processing, DNA extraction, and nanopore sequencing, was 4.5 h.

Additionally, pathogen identification for *E. coli*,* K. pneumoniae*, and *P. mirabilis* was possible at 10^3^ CFU/mL using MinION sequencing, indicating the potential of this approach at lower clinically relevant CFUs. Nanopore sequencing-based detection is also cost-effective, with the estimated sequencing cost per sample in the current study being ca. 37 €, which aligns with our previous study on sepsis diagnosis [24].

### Absorbance provides a preliminary estimate of the bacterial inoculum concentration

This study used a correlation between the A600 and the cell concentration to mimic clinically relevant UTIs using fresh urine samples at different CFU/mL concentrations. In most clinical cases, UTI is considered microbiologically confirmed when the bacterial count in urine culture is ≥ 10^5^ CFU/mL [10]. However, lower bacterial concentrations, such as 10^3^ and 10^2^ CFU/mL, might still be clinically relevant, particularly in cases of cystitis in children [35]. Our results indicate that A600 didn’t always correlate with the bacterial CFU count. Similar discrepancies have also been reported in interlaboratory studies [36–38], highlighting the poor relationship between A600 measurements and CFU counts, potentially leading to reproducibility and reliability concerns. In contrast, other studies have reported that A600 measurements directly correlate with cell concentration and could be reliable and reproducible [39, 40]. However, these studies accepted more than 50% error rates to arrive at this conclusion. To overcome this bias, the spiked suspensions in our study were always cultured. Although the colony count method effectively verifies the bacterial titer in solution, one must remember that this method only allows the enumeration of viable bacteria. Thus, a fresh overnight-grown culture was used to minimize experimental errors. After spiking the urine, it was observed that the bacteria did not show any lag phase, which is in contrast to our previous work with blood, where it took around four hours to reach the spiked concentration [24].

### DNeasy Blood & Tissue kit outperformed other tested DNA extraction kits

The QIAamp BiOstic Bacteremia DNA Kit, DNeasy Blood and Tissue Kit, and PowerFood Microbial Kit have been utilized most for isolating DNA from urine samples. Notably, the BiOstic Bacteremia DNA Kit and the Blood and Tissue Kit have been previously reported to preserve the bacterial diversity of the sample while yielding a sufficient amount of high-quality DNA for downstream sequencing [41–43]. The present study evaluated three commercial extraction kits from Qiagen: BiOstic, PowerFood, and DNeasy Blood and Tissue. The Blood and Tissue kit outperformed others regarding DNA yield, protocol length, and cost per sample. Even though this kit had lower per microlitre concentrations in some extractions, the DNA yield obtained was higher than for BiOstic as the elution volume of the former (200 µl) is four times higher than that of the latter (50 µl). Consequently, sufficient DNA was extracted from the samples within 35 min. The Blood and Tissue kit, which includes the lysozyme pre-treatment step, performed poorly for gram-positive spiked samples, especially for *E. faecalis* samples. We hypothesize that this might be due to the resistance to lysozyme exhibited by *E. faecalis* and certain Staphylococci species [44, 45]. Hence, alternative lysis methods such as bead beating or enzyme combinations of lysostaphin, lysozyme, and lyticase must be evaluated for efficient lysis of gram-positive bacteria [26]. Previous studies have also reported that the Blood and Tissue kit is best for DNA isolation from artificial human urine samples and canine urine [46, 47]. Karstens et al. [42] also reported higher DNA yield from human urine by the Blood and Tissue kit, although DNA produced by both BiOstic and Blood and Tissue kits performed well in downstream analyses. Moreover, the Blood and Tissue kit costs only 3.94 € per sample, making it slightly cheaper (ca. 10 − 20%) than the 4.29 € and 4.64 € per sample costs for PowerFood and BiOstic kits.

### Sufficiency of a 10^5^ CFU/mL spike for the detection of pathogens and ARGs in urine samples using nanopore sequencing

All the bacterial strains used in this study are common uropathogens and were selected based on their prevalence among complicated UTIs. These are standard strains, and the reference assemblies for these strains are available in public databases and our previous published studies [24, 25, 48, 49]. The three clinical in-house strains (*E. coli* INN 1, *E. coli* INN 2, *and E. coli* INN 3) were sequenced on Illumina and nanopore platforms, and hybrid assemblies are available in the public database. Therefore, the ground truth about these isolates’ pathogen ID and ARG profile is known. We successfully identified the pathogens in all nine samples at 10^5^ CFU/mL and in three samples at 10^3^ CFU/mL. The pathogen identification for both concentrations was obtained from the first 4000 nanopore reads sequenced within 10 min of sequencing. This is similar to our previously published results [24, 25]. Schmidt et al. [18] reported the identification of pathogens by MinION sequencing directly from urine with a TAT of 4 h. However, the samples used for the analysis were heavily infected clinical samples with CFU counts > 10^7^ CFU/mL.

The results from this study show that pathogen identification becomes challenging in samples with a bacterial concentration of 10^3^ CFU/mL or lower, indicating that this is probably the detection limit for the current methodology. This observation resonates with the existing literature where nanopore sequencing-based assays have a detection limit between 10^4^ and 10^2^ CFU/mL [50, 51]. Zhang et al. [19] tested the spiked and clinical urine samples and reported a pathogen detection limit of 10^3^- 10^4^ CFU/mL, determined from 2 hours of sequencing. Similarly, Deng et al. [52] determined the limit of detection to be 10^3^ CFU/mL for *E. coli* in spiked sputum samples. However, they used amplicon sequencing, a targeted sequencing method that amplifies specific DNA regions using PCR before sequencing [53].

The Flongle flow cells by ONT are a cost-effective version of the MinION flow cells and reduce the sequencing cost per run. To explore the viability of the Flongle for urine samples, we sequenced a subset of the bacterial strain spikes at three different concentrations, including 10^3^, 10^4^, and 10^5^ CFU/mL. Only pathogen identification was possible at all the spiked concentrations, which is in contrast to our previous work on blood cultures, where we showed the feasibility of identifying both pathogens and ARGs on Flongle flow cells [48]. This could be due to the much higher bacterial concentrations in blood cultures (≥ 10^8^ CFU/mL).

ONT has recently introduced the R10.4 flow cells, Q20 + kit V14 chemistry, and a new basecaller (Dorado), which they claim has decreased error rates of sequenced reads, improved read accuracies, and increased data output [54]. A study benchmarking R9.4.1/kit10 and R10/kit12 flow cell/chemistries showed that the R10.4 duplex reads base called with the super accuracy model had modal accuracy of 99.9 %, similar to Illumina reads, which had modal accuracy of 100.0 %. Modal accuracies for all the other approaches, including R9.4.1 and R.10.4.1 (with other base calling models), were > 97.0 %. However, the data yield of R10.4 flow cells (4.0 Gb) was comparatively lesser than R9.4.1 (11.0 Gb) after sequencing for 48 h. Moreover, the median N50 for R9.4.1 (19496 bp) was higher than those of R10.3 (16002 bp) and marginally lower than R10.4 (20976 bp) [55]. The data generated in this study is of high quality, with an average q-score of 11.5 and a minimum q-score of ≥ 9, above the default quality score (≥ 8) for the R10.4 flow cells.

### Diagnosing clinically relevant antibiotic-resistance genes through metagenomic sequencing depends on bacterial sequence coverage

At 10^5^ CFU/mL, seven of the total nine reference ARGs were identified within 30 min from the commencement of MinION sequencing. But, at 10^3^ CFU/mL, ARG detection was possible in only one sample after 13 h of sequencing. No ARGs were detected from any Flongle sequencing runs. The ability to identify ARGs from the metagenomic data suggests that enough sequencing depth is achieved for sufficient genome coverage of the target pathogen, as a high level of coverage is required for robust detection. MinION sequencing results indicate that a genome coverage of > 90% was achieved for *E. coli* NCTC 13441, *K. pneumoniae* CCUG 225T, *P. mirabilis* CCUG 2676T, *P. aeruginosa* CCUG 17619, and *S. aureus* NCTC 8325 strains at 10^5^ CFU/mL bacterial concentration. All the corresponding ARGs (*bla*_*CTX−M−15*_, *bla*_*CTX−M−2*_, *bla*_*SHV*_, *tet(J)*,* mepA*,* bla*_*OXA−396*_, *fosB*) were identified between 30 and 200 min of sequencing. The unidentified resistance genes *bla*_*TEM−1*_ and *tet(M)* present in strains *E. coli* INN 1 and *E. faecalis* were missed due to lower genome coverage. Similar results were observed for 10^3^ CFU/mL samples where the genome coverage was < 10% for all the samples. The same can be concluded for the Flongle results, as the coverage was far lower than the MinION results.

### Limitations

This study had some limitations. Firstly, the urine volume used for DNA extraction was 30 ml. Obtaining such a large volume of urine from critically ill patients with reduced kidney function might be challenging within a reasonable time. Secondly, we have observed difficulties extracting DNA from gram-positive bacteria *E. faecalis* samples as the DNA yield was insufficient for downstream analysis. Hence, the DNA extraction protocol must be further optimized for the effective lysis of gram-positive bacteria. Also, complicated UTIs, especially in cases of hospital-acquired UTIs, can result in polymicrobial infections. It has been reported that polymicrobial infections in UTIs are becoming more frequent and are more likely to be underreported and overlooked [56, 57]. Future research should optimize this method using actual clinical samples (including polymicrobial infections) from patients and compare it with the clinical routine methods used for diagnosing UTIs.

## Conclusion and perspectives

In conclusion, this proof-of-concept study showed that detecting the pathogen and ARGs was possible through direct DNA extraction and sequencing on MinION flow cells with a turnaround time of five hours. This method can significantly reduce the conventional TAT from sample collection to informed decision on antibiotic treatment from 48 to 72 h to 5 h, aiding in administering personalized and more effective antibiotic therapy, thereby increasing the chance of better clinical management of UTIs and preventing the spread of AMR. Moreover, to the best of our knowledge, this is the first published study that has used Flongle flow cells for direct sequencing of human urine containing pathogenic bacteria.

## Supplementary Information

Below is the link to the electronic supplementary material.

Supplementary file1 (DOCX 5.28 MB)

## Data Availability

The sequencing data presented in the study can be found through the accession number PRJEB73819 in the European Nucleotide Archive repository online using the URL: https://www.ebi.ac.uk/ena/browser/home.
