# Supplementary material for: Culture and amplification-free nanopore sequencing for rapid detection of pathogens and antimicrobial resistance genes from urine
Source: Eur J Clin Microbiol Infect Dis. 2024 Sep 16;43(11):2177–90. doi: 10.1007/s10096-024-04929-1 (PMC11534888; doi:10.1007/s10096-024-04929-1)
Supplement: Supplementary file 1 — Supplementary file1 (DOCX 5.28 MB) [file 10096_2024_4929_MOESM1_ESM.docx]

**Culture and amplification free nanopore sequencing for rapid detection of pathogens and antimicrobial resistance genes from urine**

Anurag Basavaraj Bellankimath^1^, Crystal Chapagain^1^, Sverre Branders^1^, Jawad Ali^1^, Robert C Wilson^1^, Truls E. Bjerklund Johansen^2,3^, and Rafi Ahmad^1,4*^

^1^Department of Biotechnology, Inland Norway University of Applied Sciences, Holsetgata 22, 2317, Hamar, Norway.

^2^Institute of Clinical Medicine, University of Oslo, Norway and ^3^Institute of Clinical Medicine, University of Aarhus, Denmark

^4^Institute of Clinical Medicine, Faculty of Health Sciences, UiT - The Arctic University of Norway, Hansine Hansens veg 18, 9019, Tromsø, Norway

Email: [anurag.bellankimath@inn.no](mailto:anurag.bellankimath@inn.no),[crystal.chapagain@inn.no](mailto:crystal.chapagain@inn.no),[jawad.ali@inn.no](mailto:jawad.ali@inn.no), [sverre.branders@inn.no](mailto:sverre.branders@inn.no), [robert.wilson@inn.no](mailto:robert.wilson@inn.no), [t.e.b.johansen@medisin.uio.no](mailto:t.e.b.johansen@medisin.uio.no) , [rafi.ahmad@inn.no](mailto:rafi.ahmad@inn.no)^*^

Supplementary Table 1: The list of the relevant pathogenic strains and their corresponding antibiotic resistance genes used in the study.

| **Bacterial strains** | **Reference antibiotic resistance genes (ARG’s)** |
| --- | --- |
| *E. coli* NCTC 13441 | *CTX-M-15* |
| *E. coli* INN 1 | *TEM-1* |
| *E. coli* INN 2 | *CTX-M-2* |
| *E. coli* INN 3 | *TEM-1* |
| *K. pneumoniae* CCUG225T | *SHV-11* |
| *P. mirabilis* CCUG 2676T | *catA*, *tet(J)* |
| *P. aeruginosa* CCUG 17619 | *blaOXA-396* |
| *E. faecalis* CCUG 9997 | *lsa(A)*, *tet(M)* |
| *S. aureus* NCTC 8325 | *fosB*, *mepA* |


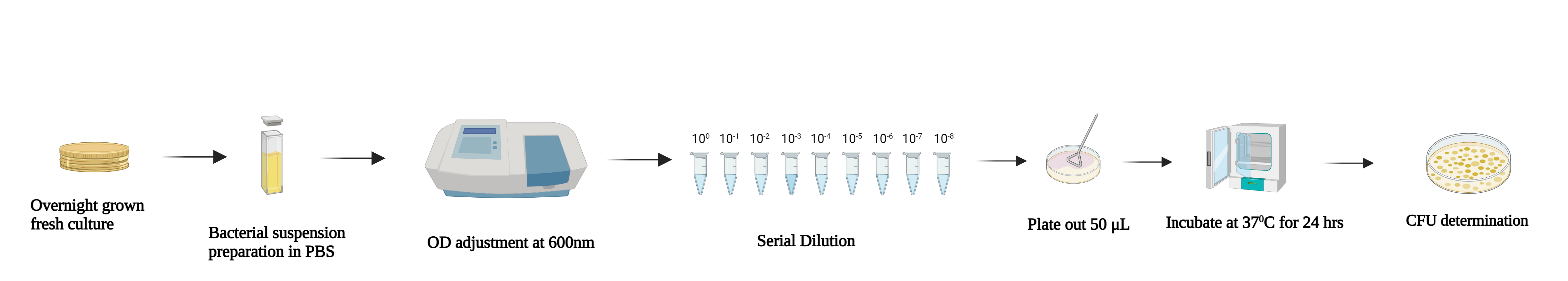


Supplementary Figure 1: A graphical overview of the different steps involved in the optical density and standard platting-based methods for inoculum optimisation. The figure was created at biorender.com.

**Supplementary Table 2**: The correlation between the absorbance (A600) and colony formation units (CFU/mL) for pathogenic strains that were used in the study.

| **Bacterial Strain** | **Absorbance (A600)** | **CFU/ml** |
| --- | --- | --- |
| *E. coli* NCTC 13441 | 1.39 | 8.0 × 10^8^ |
|  | 1.38 | 6.0 × 10^8^ |
|  | 1.31 | 6.1 × 10^8^ |
| *K. pneumoniae* CCUG 225T | 1.28 | 5.5 × 10^8^ |
|  | 1.25 | 4.7 × 10^8^ |
|  | 1.26 | 6.3 × 10^8^ |
| *S. aureus* NCTC 8325 | 1.55 | 7.8 × 10^8^ |
|  | 1.38 | 7.9 × 10^8^ |
| *S. aureus* CCUG 35600 | 1.28 | 1.0 × 10^9^ |
|  | 1.59 | 1.7 × 10^9^ |
| *P. mirabilis* CCUG 2676T | 2.1 | 1.3 × 10^9^ |
|  | 1.83 | 1.2 × 10^9^ |
|  | 2.2 | 3.3 × 10^9^ |
| *E. coli* INN 1 | 2.19 | 2.8 × 10^9^ |
| *E. coli* INN 2 | 2.12 | 3.2 × 10^9^ |
| *E. coli* INN 3 | 2.19 | 2.0 × 10^9^ |
|  | 2.12 | 2.8 × 10^9^ |
| *S. aureus* CCUG17621 | 2.25 | 6.0 × 10^8^ |
| *E. faecalis* CCUG 9997 | 1.93 | 3.5 × 10^9^ |
|  | 2.2 | 5.2 × 10^9^ |
|  | 2.22 | 4.6 ×10^9^ |
| *P. aeruginosa* CCUG 17619 | 1.9 | 1.5 × 10^9^ |
|  | 1.9 | 2.4 × 10^9^ |

Supplementary Table 3: CFU results for the urine analogue experiment. Column T_0_ denotes the time zero CFU/mL concentrations, while column T_1_ denotes the CFU one hour post spiking at 10^3^ and 10^5^ CFU/mL.

| **Bacterial Strain** | **T_0_ (Time zero)** | | **T_1_ (One hour)** | |
| --- | --- | --- | --- | --- |
|  | 10^3^ CFU/mL | 10^5^ CFU/mL | 10^3^ CFU/mL | 10^5^ CFU/mL |
| *E. coli* NCTC 13441 | 7.20 × 10^3^ | 8.40 × 10^5^ | 4.24 × 10^3^ | 7.80 × 10^5^ |
| *K. pneumoniae* CCUG 225T | NA | 3.80 × 10^5^ | 3.40 × 10^3^ | 3.40 × 10^5^ |
| *P. mirabilis* CCUG 2676T | 6.0 × 10^3^ | 5.0 × 10^5^ | 6.40 × 10^3^ | 6.40 × 10^5^ |
| *S. aureus* NCTC 8325 | 4.0 × 10^1^ | 1.80 × 10^4^ | 4.80 × 10^2^ | 1.0 × 10^4^ |
| *P. aeruginosa* CCUG 17619 | 5.0 × 10^3^ | 3.06 × 10^5^ | 5.30 × 10^3^ | 4.94 × 10^5^ |
| *E. faecalis* CCUG 9997 | 1.0 × 10^4^ | 9.40 × 10^5^ | 1.0 × 10^4^ | 1.0 × 10^6^ |


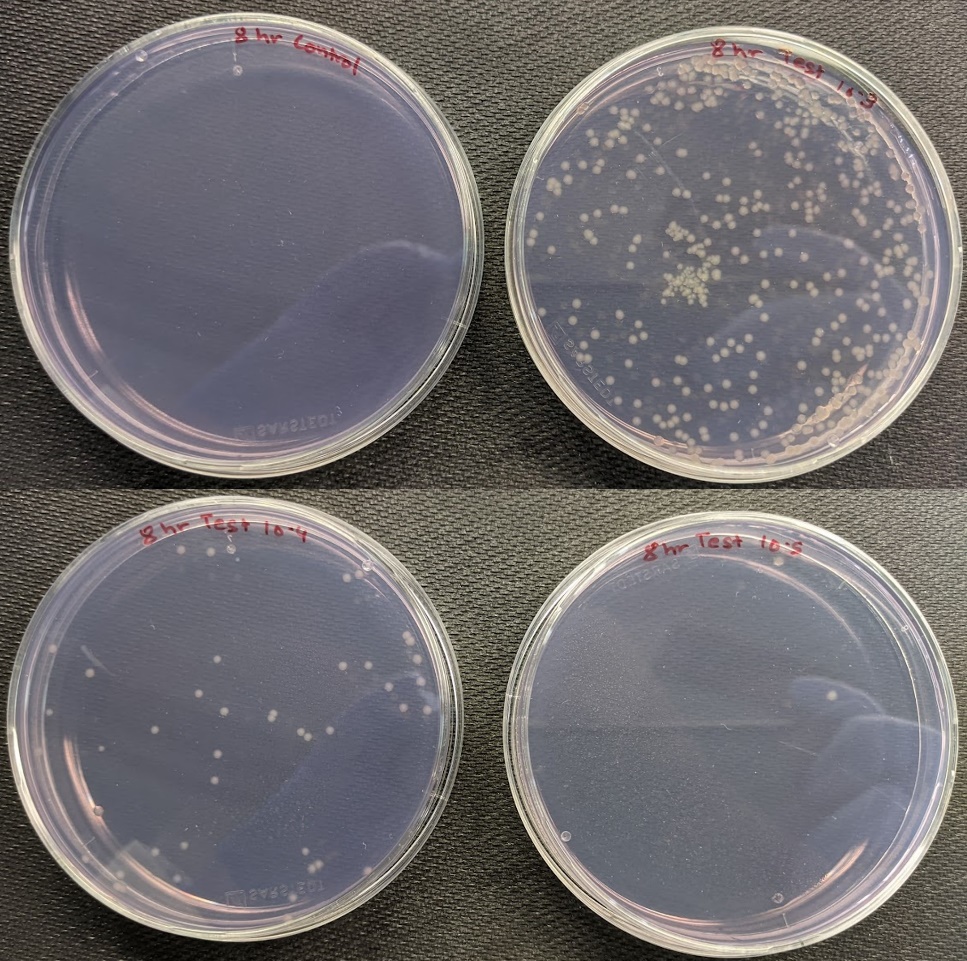

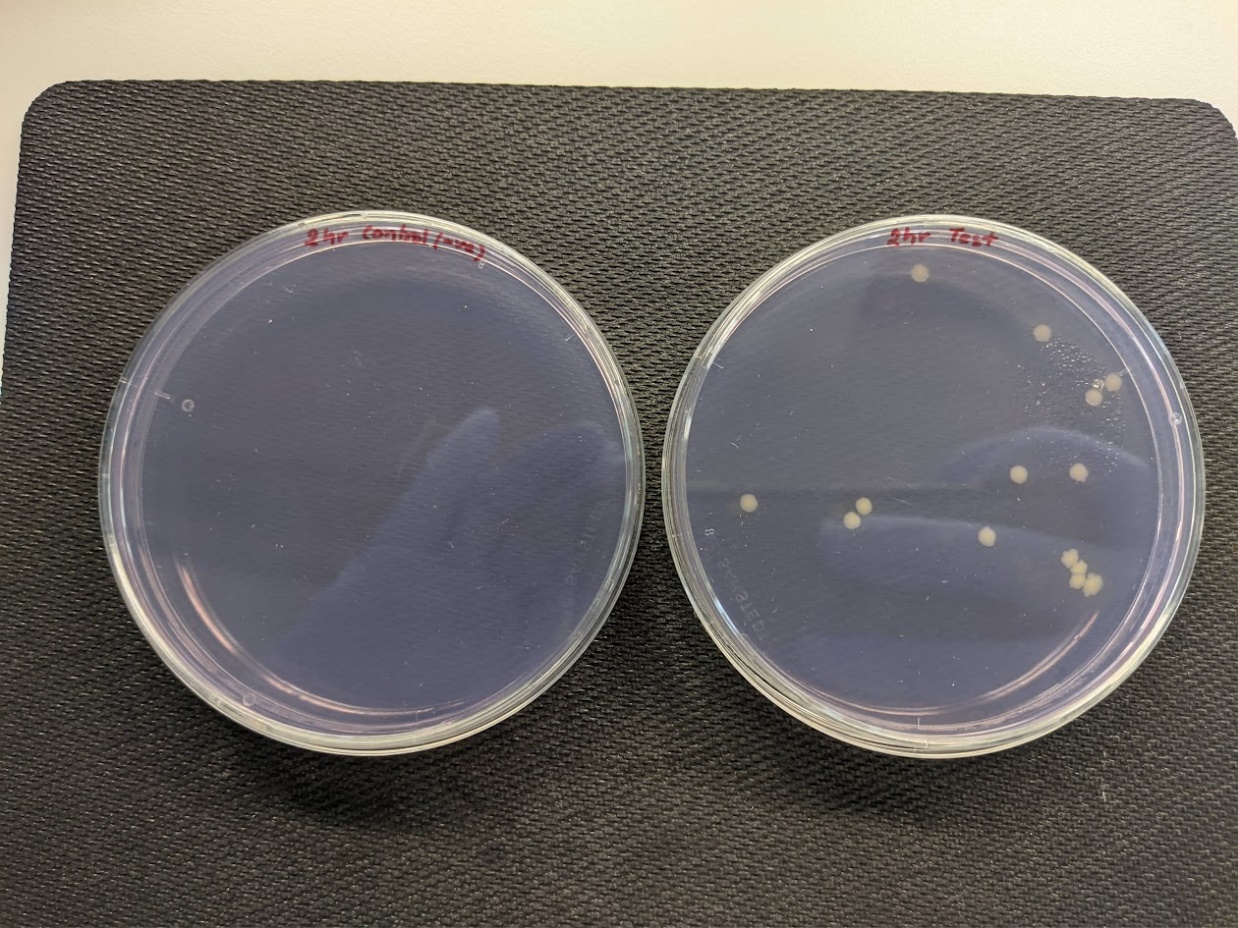


**d**

**b**

**a**

**c**

Supplementary Figure 2: Urine control experiments to determine the background flora and contaminants of the healthy urine sample. The control sample (healthy urine, subfigure a & b) and the spiked urine sample (subfigure c & d) were cultured on MacConkey agar. No bacterial growth was observed on the control plates, while the bacterial colonies were observed in the spiked samples.

Supplementary Table 4: List of the primers used for confirmation of the presence of the pathogenic DNA in the DNA extracted from the spiked samples prior to nanopore sequencing.

| **Target Species** | **Target** | **Product size** | **Primer** | **Primer Sequence** | **Reference** |
| --- | --- | --- | --- | --- | --- |
| Human | *β-Actin* | ~ 100 bp | Human-F | CGGCCTTGGAGTGTGTATTAAGTA | [1] |
|  |  |  | Human-R | TGCAAAGAACACGGCTAAGTGT |  |
| *E. coli* | *UspA* | ~ 850 bp | uspA-F | CCGATACGCTGCCAATCAGT | [1] |
|  |  |  | uspA-R | ACGCAGACCGTAGGCCAGAT |  |
| *S. aureus* | *nuc* | ~ 65 bp | Nuc-F | GGGTTGATACGCCAGAAACG | [2] |
|  |  |  | Nuc-R | TGATGCTTCTTTGCCAAATGG |  |
| *K. pneumoniae* | *Khe* | 428 bp | Khe-Kpne-F | TGATTGCATTCGCCACTGG | [3] |
|  |  |  | Khe-Kpne-R | GGTCAACCCAACGATCCTG |  |
| *P. mirabilis* | *UrePa* | 156 bp | UrePa-pmir-F | GGTGAGATTTGTATTAATGG | [4] |
|  |  |  | UrePa-pmir-R | ATAATCTGGAAGATGACGAG |  |
| *E. faecalis* | *GroEs* | 185 bp | EfGroEs-F | GGAATTGTTCTTGCATCCGT | [5] |
|  |  |  | EfGroEs-R | ACAATTAAGTATTCTACGCC |  |
| *P. aureginosa* | *phzA2* | 325bp | PA2-F | GTTTACCGACAACCTGGAA | [6] |
|  |  |  | PA2-R | GCAATAGCCCTGCGGATAC |  |


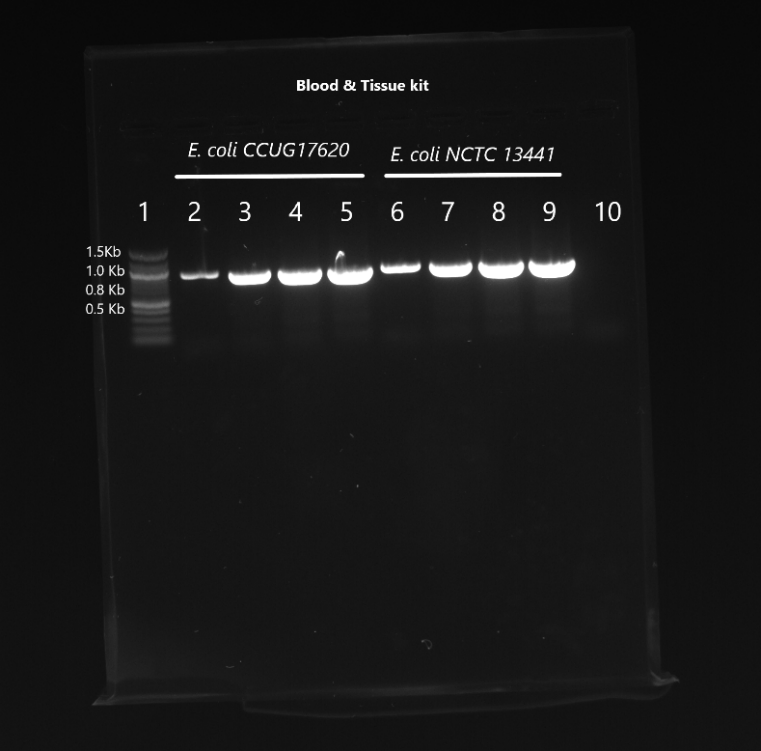

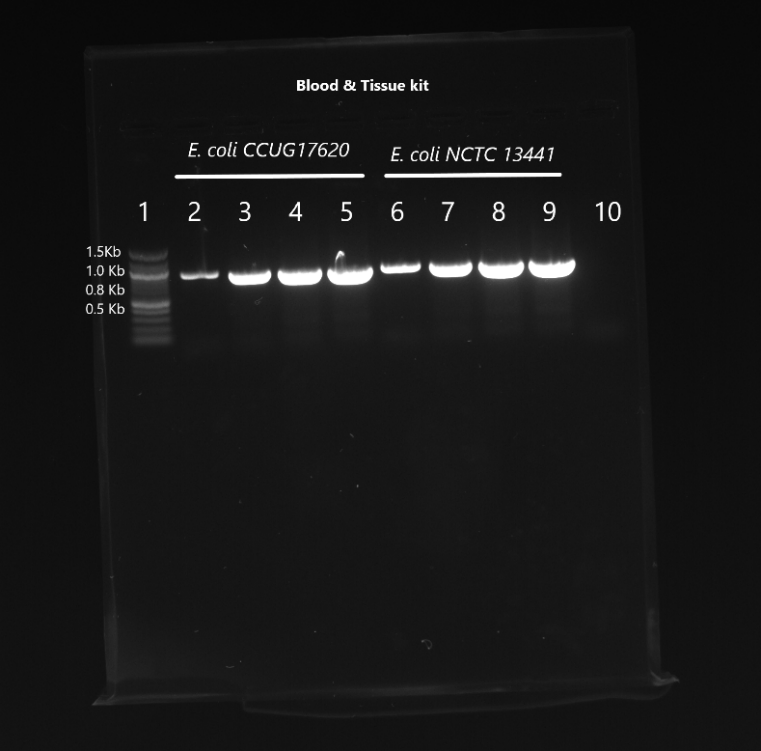

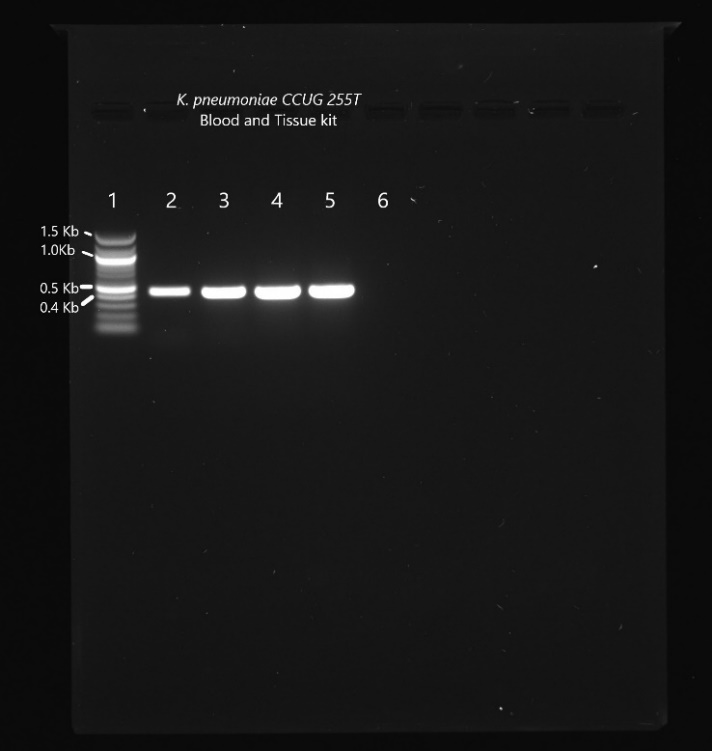

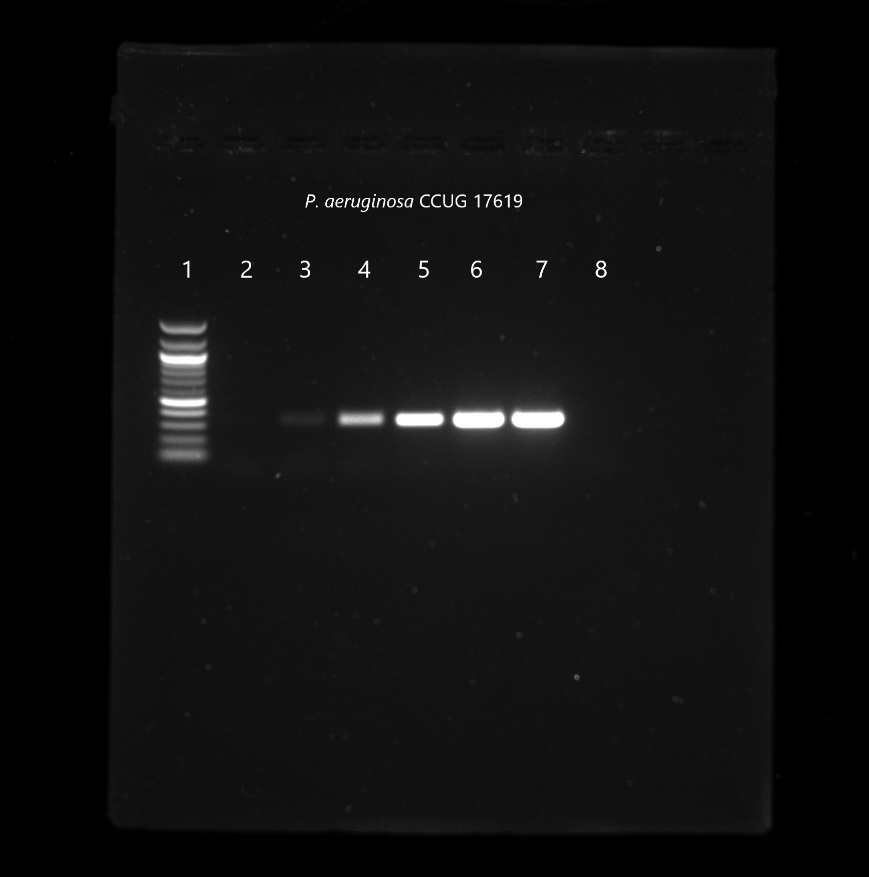


**a**

**b**

**d**

**
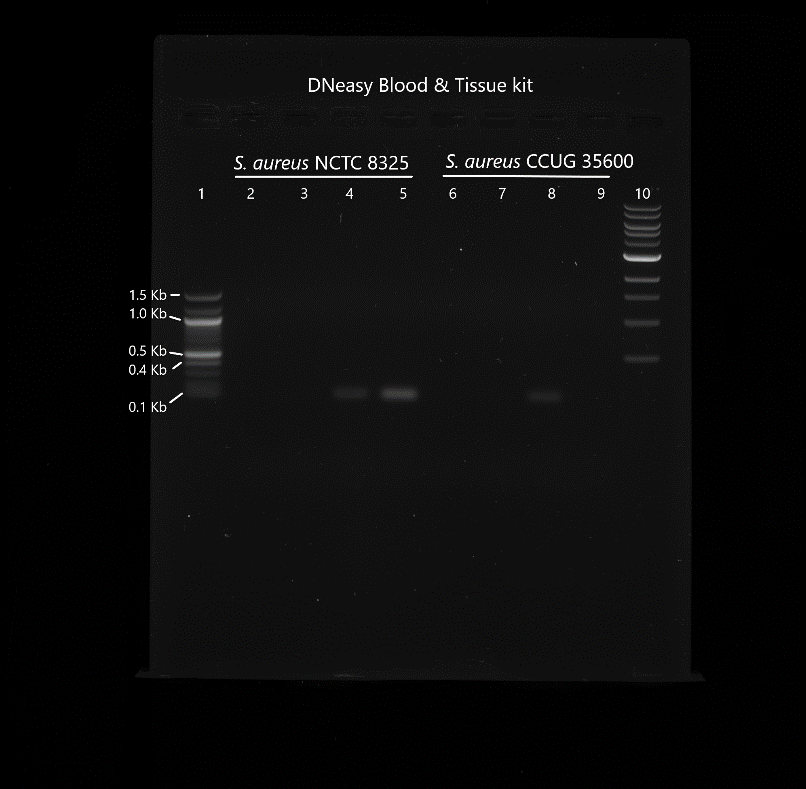
**

**c**


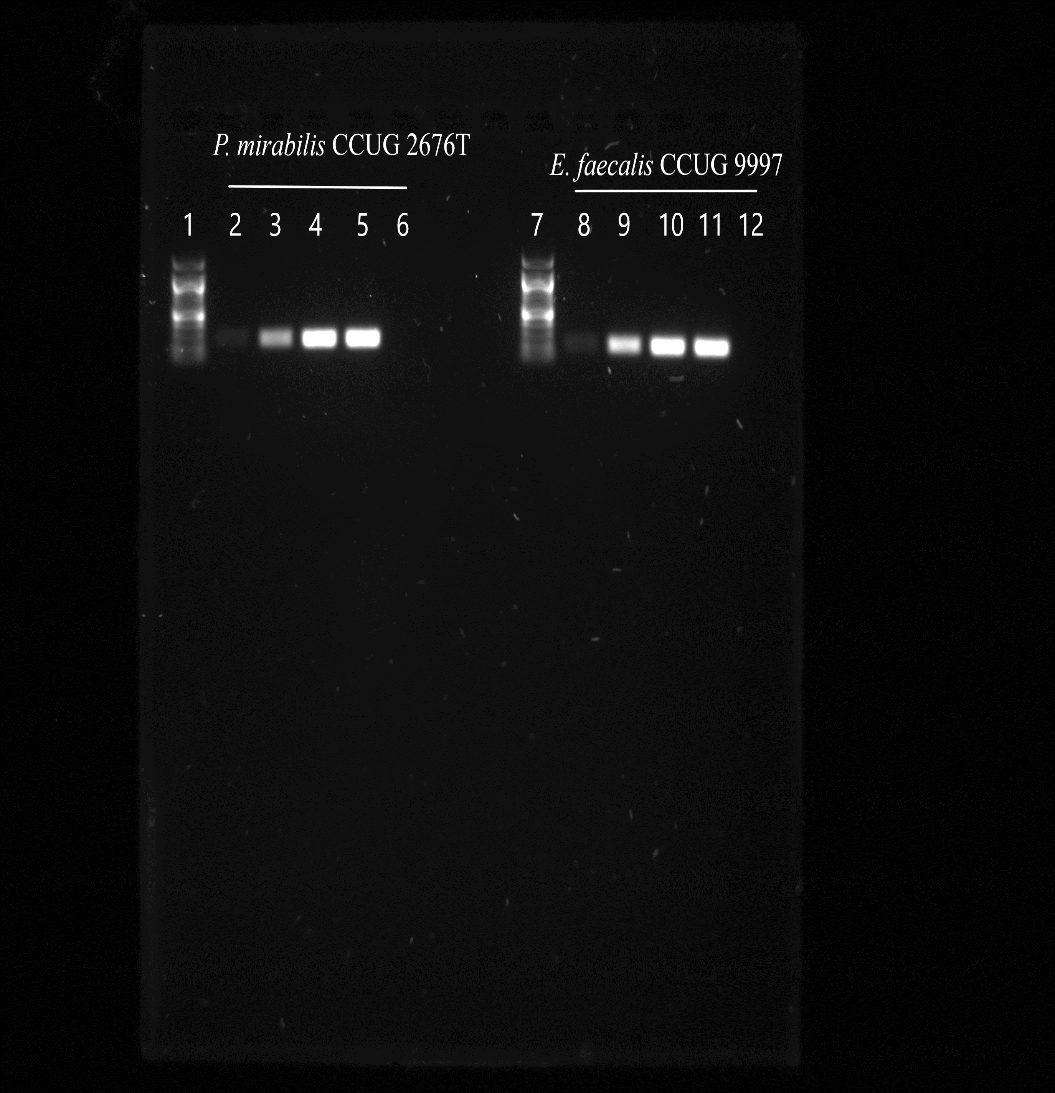

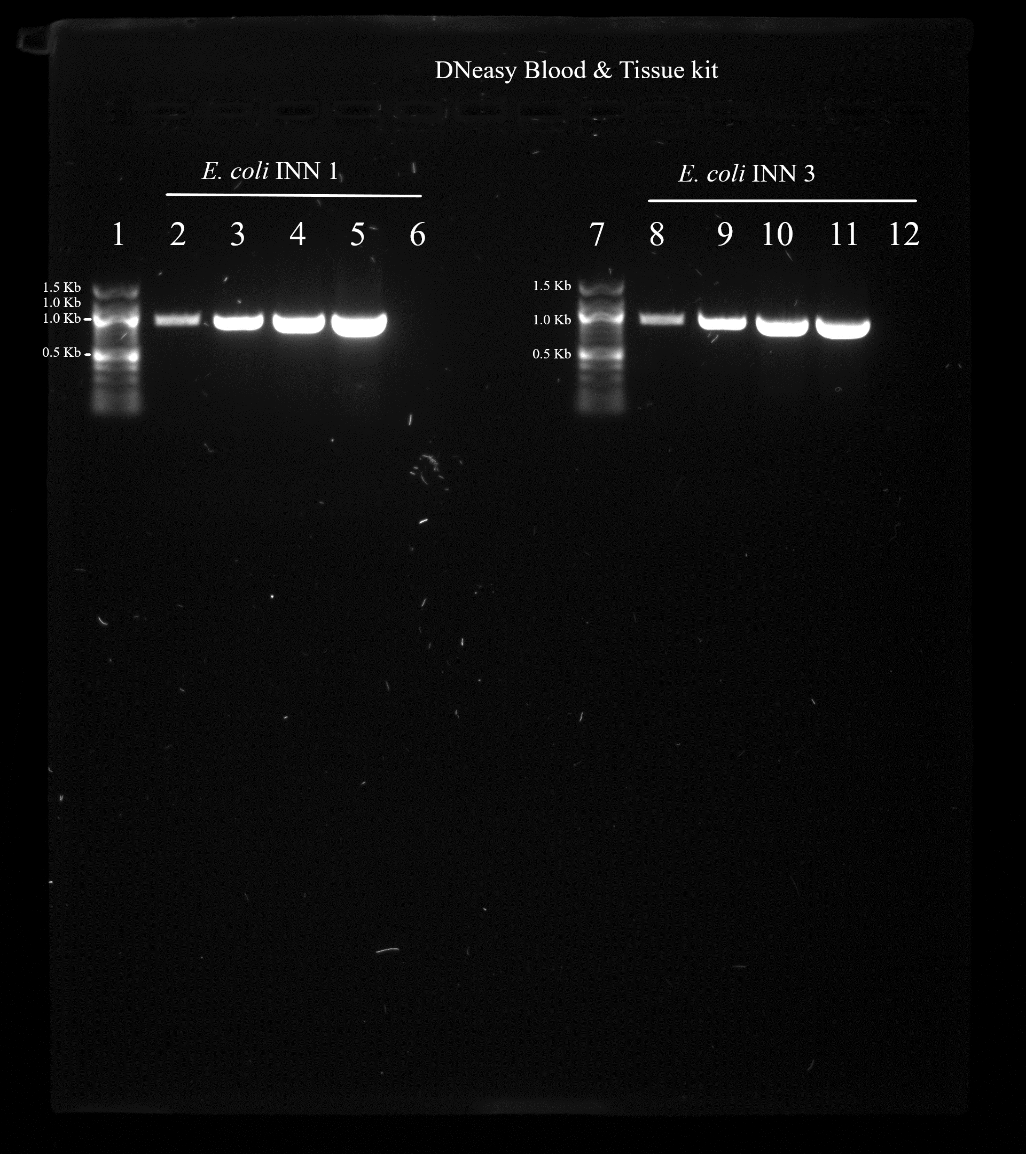

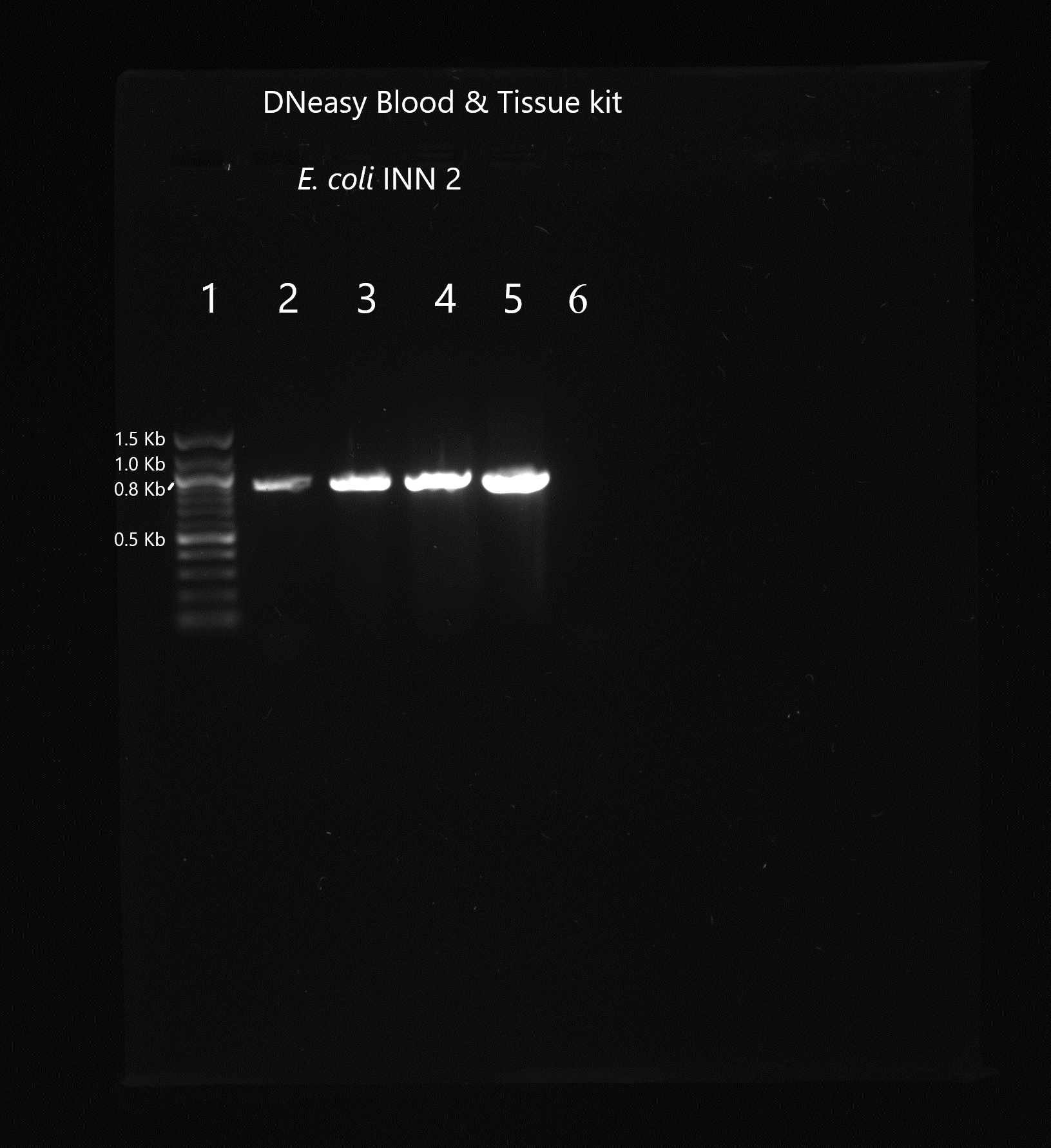


**g**

**f**

**e**

Supplementary Figure 3: PCR confirmation of the presence of spiked pathogens in the DNA extracted directly from the spiked urine samples. The amplification was performed using the respective housekeeping genes.

subfigure (a) Samples spiked with *E. coli* CCUG 17620 and *E. coli* NCTC 13441. Identification was performed using gene *UspA* (850 bp). Lane description: 1: 100bp NEB DNA ladder, 2: 10^2^ CFU/mL, 3: 10^3^ CFU/mL, 4: 10^4^ CFU/mL, 5: 10^5^ CFU/mL, 6: 10^2^ CFU/mL, 7: 10^3^ CFU/mL, 8: 10^4^ CFU/mL, 9: 10^5^ CFU/mL, 10: Negative template control.

subfigure (b) Samples spiked with *K. pneumoniae* CCUG 225T. Identification was performed using gene *Khe* (428 bp). Lane description: 1: 100bp NEB DNA ladder, 2: 10^2^ CFU/mL, 3: 10^3^ CFU/mL, 4: 10^4^ CFU/mL, 5: 10^5^ CFU/mL, 6: Negative template control.

subfigure (c) Samples spiked with *S. aureus* NCTC 8325 and *S. aureus* NCTC 8325. Identification was performed using gene *nuc* (~ 65 bp). Lane description: 1: 100bp NEB DNA ladder, 2: 10^2^ CFU/mL, 3: 10^3^ CFU/mL, 4: 10^4^ CFU/mL, 5: 10^5^ CFU/mL, 6: 10^2^ CFU/mL, 7: 10^3^ CFU/mL, 8: 10^4^ CFU/mL, 9: 10^5^ CFU/mL, 10: 1kB DNA ladder.

subfigure (d) Samples spiked with *P. aeruginosa* CCUG 17619. Identification was performed using gene *phzA2* (~ 325 bp). Lane description: 1: 100bp NEB DNA ladder, 2: 10^2^ CFU/mL, 3: 10^3^ CFU/mL, 4: 10^4^ CFU/mL, 5: 10^5^ CFU/mL, 8: negative template control.

subfigure (e) Samples spiked with *E. coli* INN 2. Identification was performed using gene *UspA* (850 bp). Lane description: 1: 100bp NEB DNA ladder, 2: 10^2^ CFU/mL, 3: 10^3^ CFU/mL, 4: 10^4^ CFU/mL, 5: 10^5^ CFU/mL, 6: negative template control.

**subfigure (f)**. Samples spiked with *E. coli* INN 1 & *E. coli* INN 3. Identification was performed using gene *UspA* (850 bp). Lane description: 1: 100bp NEB DNA ladder, 2: 10^2^ CFU/mL, 3: 10^3^ CFU/mL, 4: 10^4^ CFU/mL, 5: 10^5^ CFU/mL, 6: negative template control, 7: 100bp NEB DNA ladder, 8: 10^2^ CFU/mL, 9: 10^3^ CFU/mL, 10: 10^4^ CFU/mL, 11: 10^5^ CFU/mL, 12: negative template control

**subfigure (g)**. Samples spiked with *P. mirabilis* 2676T & *E. faecalis* CCUG 9997. Identification was performed using the respective housekeeping genes *UrePa* (156 bp) and *GroES* (185 bp). Lane description: 1: 100bp NEB DNA ladder, 2: 10^2^ CFU/mL, 3: 10^3^ CFU/mL, 4: 10^4^ CFU/mL, 5: 10^5^ CFU/mL, 6: negative template control, 7: 100bp NEB DNA ladder, 8: 10^2^ CFU/mL, 9: 10^3^ CFU/mL, 10: 10^4^ CFU/mL, 11: 10^5^ CFU/mL, 12: negative template control


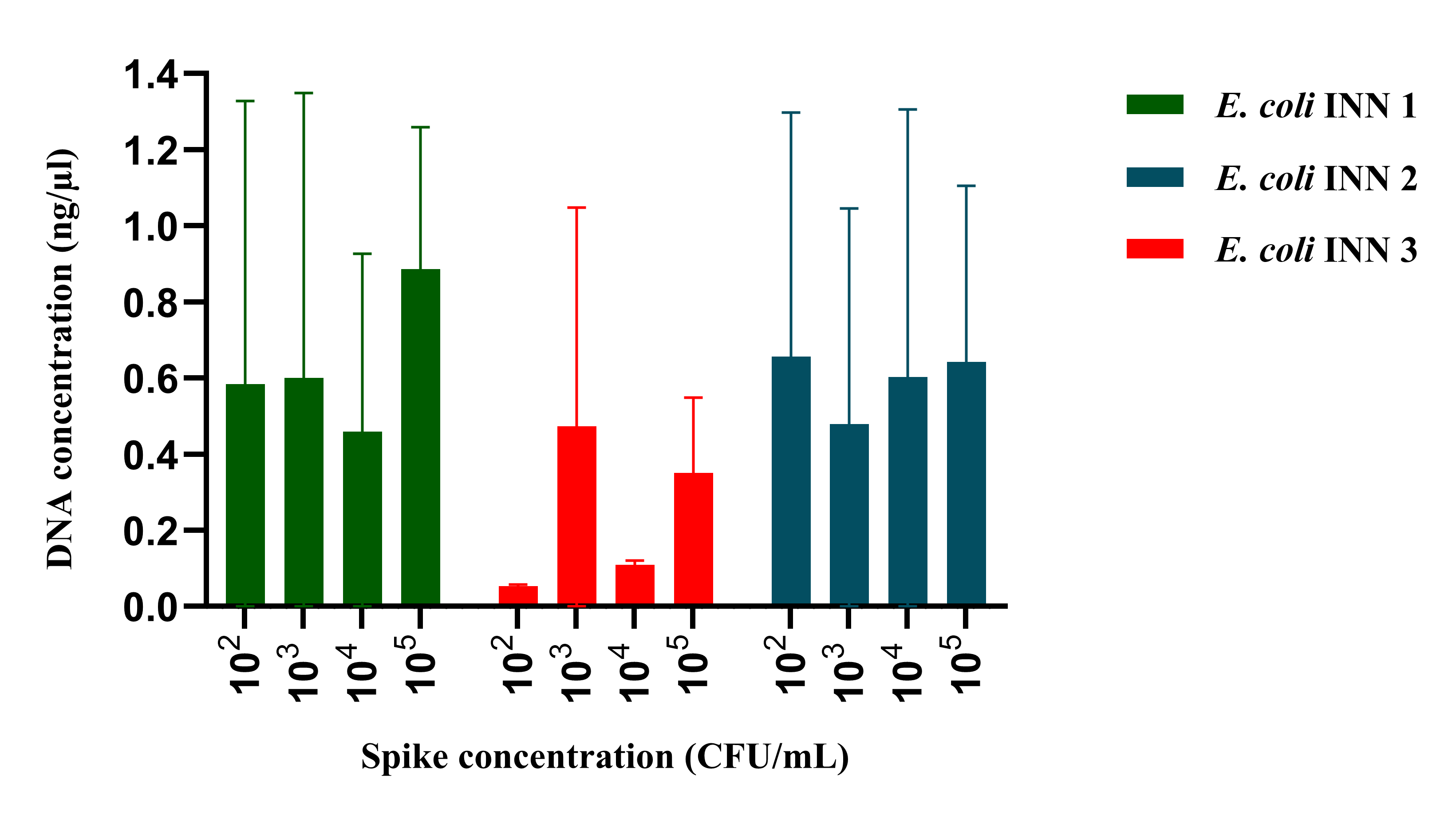


Supplementary Figure 4: Comparison of the extracted DNA concentration between the three internal strains *E. coli* INN 1, *E. coli* INN 2 and *E. coli* INN 3. The DNA extractions were performed using the Blood and Tissue kit in duplicates at the spike concentrations of 10^2^, 10^3^, 10^4^ and 10^5^ CFU/mL. The figure was created in GraphPad Prism (10.0.2).


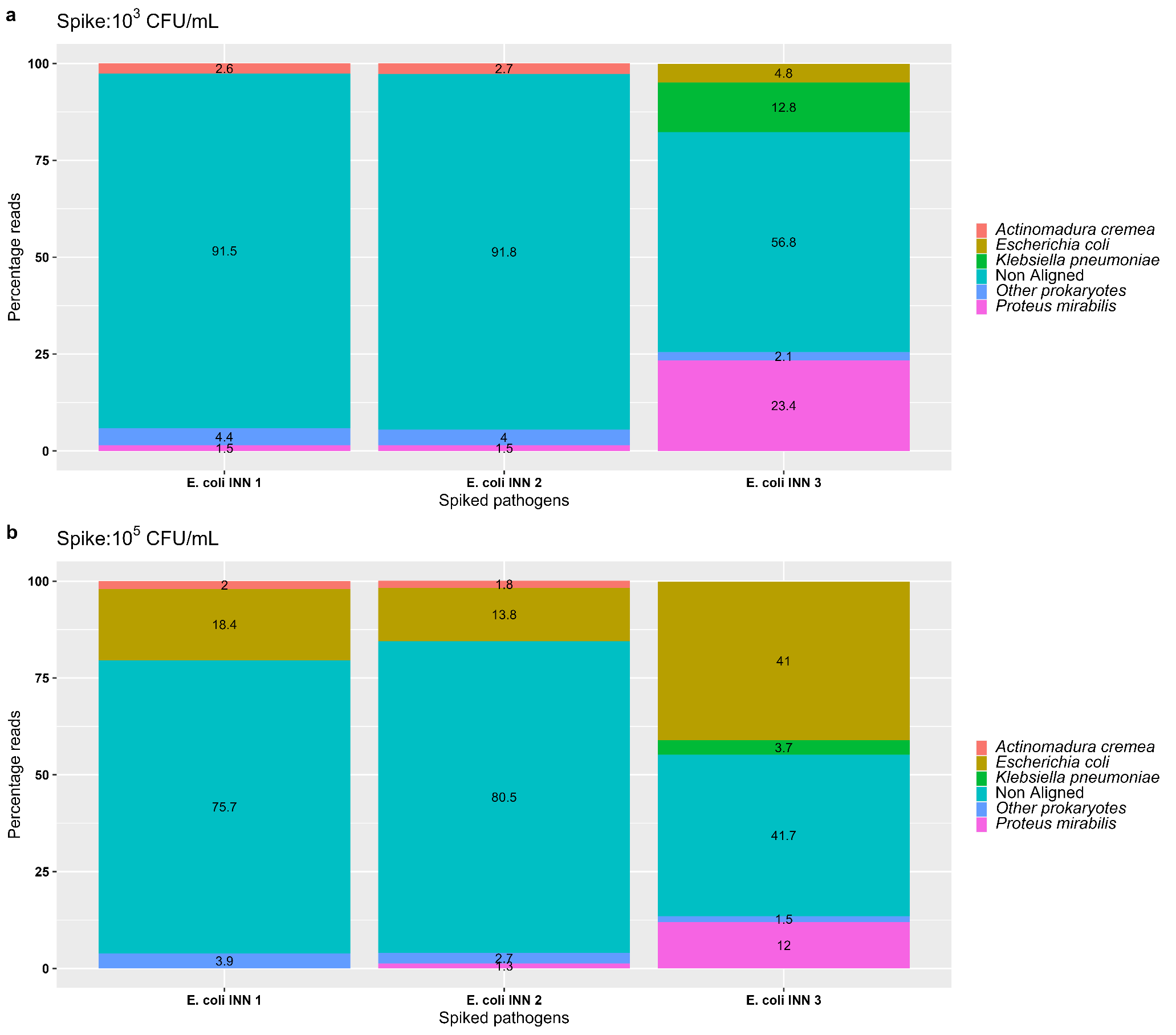


Supplementary Figure 5: Sequencing results for the three internal test strains *E. coli* INN 1, *E. coli* INN 2 and *E. coli* INN 3. Subfigure 4. a represents the results from the 10^3^ CFU/mL spiked samples, while subfigure (b) denotes the results from 10^5^ CFU/mL spiked samples. The denoted percentage reads are based on the BLAST search against the RefProk database (prokaryotic sequence data only). The figure was created in R studio (1.4.1106).


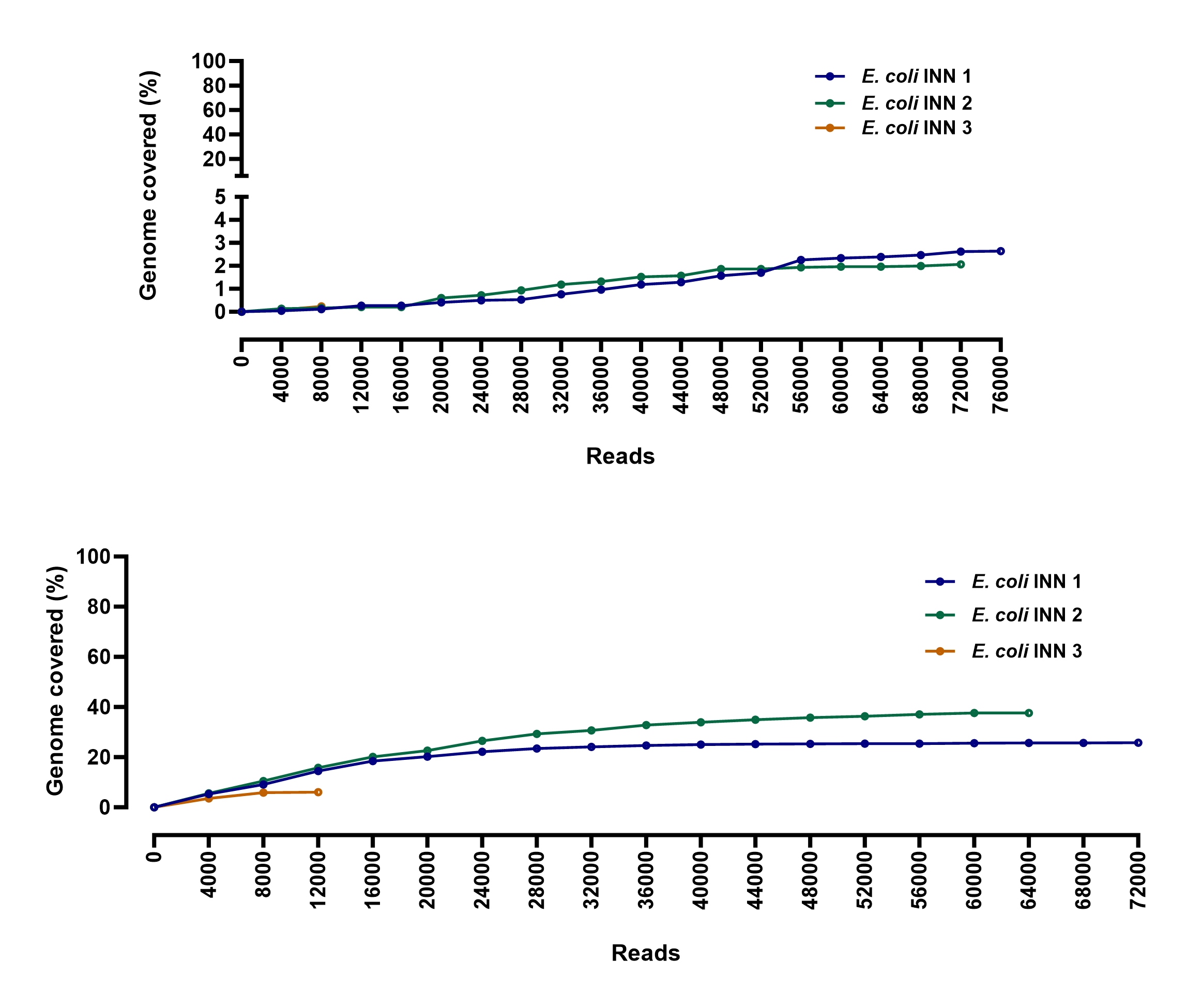


**b**

**a**

Supplementary Figure 6: Genome coverage of the three target species, *E. coli* INN 1, *E. coli* INN 2, and *E. coli* INN 3, based on the sequencing reads generated during the entire sequencing run. Subfigure (a) represents the pathogen coverage obtained for the samples spiked with 10^3^ CFU/mL concentration, while subfigure (b) is the pathogen coverage from the samples spiked with a pathogen concentration of 10^5^ CFU/mL. The figure was created in GraphPad Prism (10.0.2).

**References:**

1. Ali J, Joshi M, Ahmadi A, Str KO, Ahmad R. Increased growth temperature and vitamin B12 supplementation reduces the lag time for rapid pathogen identification in BHI agar and blood cultures [version 2; peer review: 2 approved]. 2023;

2. Ahmadi A, Khezri A, Nørstebø H, Ahmad R. A culture-, amplification-independent, and rapid method for identification of pathogens and antibiotic resistance profile in bovine mastitis milk. Frontiers in Microbiology [Internet] 2023 [cited 2023 10];13. doi: https://www.frontiersin.org/articles/10.3389/fmicb.2022.1104701

3. Hua Wu YS. Detection and Analysis of Regional Trends of Klebsiella pneumonia Causing Liver Abscess. Clin Microbiol [Internet] 2015 [cited 2023 20];04. doi: http://www.esciencecentral.org/journals/detection-and-analysis-of-regional-trends-of-klebsiella-pneumonia-causingliver-abscess-2327-5073-1000208.php?aid=56943doi: 10.4172/2327-5073.1000208

4. Zhang W, Niu Z, Yin K, Liu P, Chen L. Quick identification and quantification of Proteus mirabilis by polymerase chain reaction (PCR) assays. Ann Microbiol 2013;63:683–9. doi: 10.1007/s13213-012-0520-x

5. Teng L-J, Hsueh P-R, Wang Y-H, Lin H-M, Luh K-T, Ho S-W. Determination of *Enterococcus faecalis groESL* Full-Length Sequence and Application for Species Identification. J Clin Microbiol 2001;39:3326–31. doi: 10.1128/JCM.39.9.3326-3331.2001

6. Wang C, Ye Q, Jiang A, Zhang J, Shang Y, Li F, et al. Pseudomonas aeruginosa Detection Using Conventional PCR and Quantitative Real-Time PCR Based on Species-Specific Novel Gene Targets Identified by Pangenome Analysis. Front. Microbiol. 2022 4;13:820431. doi: 10.3389/fmicb.2022.820431
